# DNA replication stress and translational repression converge to drive CDK1- and caspase-dependent apoptosis in Ewing sarcoma

**DOI:** 10.1038/s41388-026-03845-2

**Published:** 2026-06-10

**Authors:** Stacia L. Koppenhafer, Mary V. Thomas, Mian T. Mhindu, Jessica A. O. Zimmerman, David J. Gordon

**Affiliations:** Department of Pediatrics, Division of Pediatric Hematology/Oncology, University of Iowa, Iowa City, Iowa USA

**Keywords:** Sarcoma, Cancer therapy

## Abstract

Despite aggressive multimodal therapy, including cytotoxic chemotherapy, surgery, and radiation, the prognosis for patients with Ewing sarcoma remains poor, particularly for those with metastatic or relapsed disease. Combining agents that increase DNA replication stress with ATR-CHK1-WEE1 pathway inhibitors, which disrupt the DNA damage response and cell cycle checkpoints, is a promising strategy under clinical investigation in Ewing sarcoma and other cancers. However, the mechanisms by which these drug combinations selectively kill cancer cells under replication stress remain incompletely understood and are often attributed, without strong supporting evidence in many tumor types, to forced mitotic entry. In this study, we show that inhibition of the ATR-CHK1-WEE1 pathway in S-phase-arrested Ewing sarcoma cells triggers rapid apoptosis within 2–4 h, without widespread mitotic entry. This apoptotic response is driven by the activation of cyclin-dependent kinase 1 (CDK1) and is caspase-dependent. We further show that dual targeting of DNA replication and ATR-CHK1-WEE1 signaling in Ewing sarcoma tumors suppresses protein synthesis, and inhibition of protein synthesis prevents cell cycle progression and premature mitotic entry—providing a mechanistic explanation for why aberrant CDK1 activation does not drive mitosis in this context. Moreover, while apoptosis is induced rapidly following drug treatment, the suppression of protein synthesis is prolonged and persists beyond drug removal, suggesting distinct early and late mechanisms of drug-induced toxicity. Collectively, these findings define a unique CDK1- and caspase-dependent apoptotic pathway in response to replication stress and offer new insights into the molecular basis of this therapeutic vulnerability in Ewing sarcoma.

## Introduction

DNA replication stress is a hallmark of many cancers and arises when cells encounter obstacles during DNA synthesis, leading to stalled replication forks, genomic instability, and activation of checkpoint signaling [1, 2]. In Ewing sarcoma—a highly aggressive pediatric and young adult bone and soft tissue cancer—multiple mechanisms contribute to elevated baseline replication stress [1]. These include cell cycle dysregulation, increased R-loop formation, elevated expression of the helicase SLFN11, loss of ATM activity, EWSR1 haploinsufficiency due to the EWS::FLI1 fusion, and a BRCA1-deficient phenotype [3–12]. Collectively, these features create a dependency on replication stress response pathways, rendering Ewing sarcoma cells particularly vulnerable to agents that either amplify replication stress or inhibit its resolution [13–20].

Ribonucleotide reductase (RNR), the rate-limiting enzyme in deoxyribonucleotide synthesis, plays a central role in maintaining replication fidelity. Inhibition of either the RRM1 or RRM2 subunit disrupts dNTP pools, impairs S-phase progression, and induces replication stress [21]. The ATR-CHK1-WEE1 signaling axis orchestrates the cellular response to replication stress and DNA damage by regulating origin firing, fork stability, and cell cycle checkpoints [22, 23]. Notably, dual inhibition of RNR and the ATR-CHK1-WEE1 pathway has shown synergistic anti-tumor activity in vitro and in vivo, including in xenograft models of Ewing sarcoma and other malignancies [14, 17–19, 24–27]. Multiple inhibitors targeting these pathways are currently undergoing clinical evaluation, both as monotherapies and in combination regimens [23].

Despite promising preclinical and clinical data, the molecular mechanisms underlying the selective cytotoxicity of these agents—and the pathways that mediate resistance—remain incompletely understood. The prevailing model suggests that ATR-CHK1-WEE1 inhibitors kill cancer cells by forcing premature mitotic entry, which can cause chromosomal mis-segregation, micronuclei formation, and cell death [26, 28–34]. However, this model lacks robust mechanistic validation in Ewing sarcoma tumors. In this study, we identify a distinct mechanism of cell death in Ewing sarcoma cells exposed to ATR-CHK1-WEE1 inhibition during S-phase. We show that these cells undergo rapid apoptosis within 2–4 h, independent of widespread mitotic entry. This apoptotic response is driven by aberrant activation of cyclin-dependent kinase 1 (CDK1) and is caspase-dependent. Furthermore, we demonstrate that dual targeting of DNA replication and checkpoint signaling suppresses protein synthesis, and that inhibition of translation prevents cell cycle progression and mitotic entry—providing a mechanistic explanation for why CDK1 activation does not drive mitosis under these conditions.

Overall, these findings reveal a previously unrecognized CDK1- and caspase-dependent apoptotic mechanism and offer new insights into the molecular basis of therapeutic vulnerability in Ewing sarcoma. Importantly, this work highlights a critical interplay between DNA replication stress and protein synthesis, demonstrating that translational repression is a critical mediator of cell fate. Suppression of protein synthesis following dual inhibition of ribonucleotide reductase and ATR-CHK1-WEE1 signaling prevents cell cycle progression and mitotic entry, thereby uncoupling CDK1 activation from mitotic catastrophe and redirecting cells toward apoptosis. Together, these findings challenge existing models and provide a mechanistic framework for optimizing replication stress–based therapies in Ewing sarcoma and other tumors.

## Materials and methods

### Sex as a biological variable

Our study examined xenograft tumors growing in female mice. It is unknown whether the findings are relevant for male mice.

### Cell lines and culture

The TC71 (RRID:CVCL_2213), EW8 (RRID:CVCL_1658), SKNEP (RRID:CVCL_0631), and A673 (RRID:CVCL_0800) cell lines were provided by Dr. Kimberly Stegmaier (Dana-Farber Cancer Institute, Boston, MA). The AGPN (RRID:CVCL_X981) and TTC466 (RRID:CVCL_A444) cell lines were obtained from the Childhood Cancer Repository (Children’s Oncology Group). The ES6 (RRID:CVCL_1202) and ES1 (CVCL_1198) cell lines were obtained from the Childhood Solid Tumor Network at St. Jude Children’s Research Hospital. The HEK-293T (RRID:CVCL_1926), U2OS (RRID:CVCL_0042), and RD (RRID:CVCL_1649) cell lines were obtained from ATCC. The cell lines were maintained at 37 °C in a 5% CO_2_ atmosphere and grown in Dulbecco’s Modified Eagle’s Media (DMEM) supplemented with 10% FBS, 100 IU ml^−1^ penicillin and 100 µg ml^−1^ streptomycin. DNA fingerprinting confirmation of cell lines was performed using the short tandem repeat method and cell lines were used within 8–10 passages of thawing. Cell lines are routinely tested for mycoplasma upon acquisition and every six months thereafter (ThermoFisher Scientific, Mycoplasma Detection Kit, M7006).

### Chemical compounds

Gemcitabine (HY-17206), prexasertib (HY-18174), rabusertib (HY-14720), AZD-1775 (HY-10993), RP-6306 (HY-145817A), staurosporine (HY-15141), RO-3306 (HY-12529), AZD5438 (HY-10012), roscovitine (HY-30237), Z-VAD-FMK (HY-16658B), Q-VD-OPh (HY-12305), emricasan (HY-10396) were obtained from MedChemExpress. Doxycycline (J67043-AE), puromycin (J67236.8EQ), thymidine (A11493-06), and cycloheximide (J660-1-03) were obtained from ThermoFisher Scientific.

### Cell viability assay

Cell viability was measured using the CellTiter-Glo luminescence assay (Promega, G9241), as previously described [35, 36]. Approximately 5000 cells were plated per well of a 96-well plate, after which the cells were exposed to a range of drug concentrations for 72 h. Luminescence readings were then obtained after adding the CellTiter-Glo reagent using a FLUOstar Omega microplate reader (BMG Labtech). IC50 values were calculated using log-transformed and normalized data (GraphPad Prism 10.1.0; RRID:SCR_002798).

### Clonogenic assay

Clonogenic assays were performed as described [16]. Cells were plated in 6-well plates in triplicate, treated with drug or vehicle, and then cultured for 14 days. Colonies were then stained with crystal violet and counted using an inverted Olympus CKX41 microscope.

### Protein isolation and immunoblotting

Protein loading for the immunoblots was normalized using cell number. Protein extracts for immunoblotting were prepared by incubating cells in RIPA buffer (Boston BioProducts) that was supplemented with protease and phosphatase inhibitors (Halt Protease & Phosphatase Inhibitor Cocktail, EDTA-free; ThermoFisher Scientific) for 20 min. Supernatants were collected following centrifugation, and SDS-PAGE was used to separate proteins, which were then transferred to polyvinylidene difluoride membranes (Millipore). Membranes were cut and divided, using a pre-stained protein ladder (ThermoFisher Scientific) for molecular weight landmarks, prior to hybridization with antibodies. Antibodies to the following proteins were used in the immunoblots: actin (Cell Signaling Technology, #4970, 1:5000; RRID:AB_2223172), FLI1 (Abcam, #ab133485, 1:1000, RRID:AB_2722650), alpha tubulin (Proteintech Cat #66031-1-Ig, 1:5000, RRID:AB_11042766), RRM1 (Cell Signaling Technology, #8637, 1:1000, RRID:AB_10896851), CHK1 (Cell Signaling Technology, #2360, 1:1000, RRID:AB_11217623), p-CHK1-S345 (Cell Signaling Technology, #2348, 1:1000, RRID:AB_331212), PARP (Cell Signaling Technology, #9352, 1:1000, RRID:AB_659884), γH2AX (Cell Signaling Technology, #9718, 1:5000, RRID:AB_2118009), cleaved caspase-7 (Cell Signaling Technology, #9491, 1:1000, RRID:AB_2068144), CDK1 (Cell Signaling Technology, #9116, 1:2000, RRID:AB_2074795), CDK2 (Millipore, 05-596, 1:500, RRID:AB_2291613), p-CDK1/2 (Cell Signaling Technology, #4539, 1:1000, RRID:AB_560953), cyclin B1 (Cell Signaling Technology, #12231, 1:1000, RRID:AB_2783553), cyclin D1 (Cell Signaling Technology, #55506, 1:1000, RRID:AB_282737), cyclin E1 (Cell Signaling Technology, #20808, 1:1000, RRID:AB_2783554), and puromycin (Developmental Studies Hybridoma Bank, PMY-2A4, RRID:AB_2619605). Immunoblots were analyzed and quantified using Fiji (RRID:SCR_002285).

### siRNA transfection

Cells (1.5–3 × 10^5^) were plated one day prior to transfection in six-well plates. Cells were transfected with siRNA using Lipofectamine RNAiMax (ThermoFisher Scientific) according to the manufacturer’s instructions. siControl was obtained from Cell Signaling Technology (#6568). siCDK1 was obtained from Dharmacon (ON-TARGETplus).

### TO-CHK1-KO cell line

A codon-optimized CHK1 gene construct was obtained as a gene block (IDT; Coralville, IA) and inserted into the Lenti-X-Tet-One vector (Takara Biology) using NEBuilder HiFi DNA Assembly (NEB). After verification by sequencing, the plasmid was used to make lentivirus, as described in a previous publication [16]. CRISPR/Cas9-mediated knockout of CHK1 was then performed using a pLV[CRISPR] plasmid (VectorBuilder) that co-expresses Cas9, two gRNA (GGGCAAAGGACAGTCCGGTG and GAAGGGCGGCCGGTAGAGTA) targeting *CHK1*, and a hygromycin resistance gene. Cells were selected in hygromycin starting 48 h after viral transduction. The knockout cell lines were then single-cell cloned using flow cytometry (Becton Dickinson FACS Aria). Knockout of CHK1 in clones was then validated using immunoblotting.

### CDK2-KO cell line

CRISPR/Cas9-mediated knockout of CDK2 was performed using a pLentiCRISPR v2 plasmid (GenScript; Piscataway, NJ) that co-expresses Cas9 and a gRNA (TGTTCGTACTTACACCCATG) targeting CDK2. Lentivirus was prepared as described in previous publications, and cells were selected in 1 μg/mL puromycin starting 48 h after transduction [16]. The knockout cell lines were then single-cell cloned using flow cytometry (Becton Dickinson FACS Aria). Knockout of CDK2 was then validated using immunoblotting.

### U2OS-EWS::FLI1 cell line

A U2OS osteosarcoma cell line that expresses doxycycline-inducible EWS::FLI1 was generated by cloning EWS::FLI1-T2A-zsGreen into a pLV-TRE3G plasmid (VectorBuilder). The pLVX-EF1a-Tet3G vector (Takara Biology) was used to express the Tet-On 3G transactivator protein from the human EF1 alpha promoter. Lentivirus was prepared as described above. U2OS osteosarcoma cells were sequentially infected and selected with geneticin 500 μg/mL (pLVX-EF1a-Tet3G) and then expression of zsGreen by flow cytometry (TRE3G-EWS::FLI1-T2A-zsGreen).

### Thymidine double block

Cells were treated for 18 h overnight with thymidine (2 mM). The thymidine was then removed by washing the cells with pre-warmed 1x PBS. Fresh medium was then added, and the cells were incubated for 9 h in a tissue culture incubator at 37 °C. The cells were then treated with a second round of thymidine (2 mM) for another 18 h at 37 °C.

### Fluorescent cell cycle reporter

A lentiviral (pLV) plasmid expressing the PIP-FUCCI fluorescent indicator protein construct was obtained from VectorBuilder [37]. Lentivirus was prepared as described in previous publications, and fluorescent cells were isolated using flow cytometry (Becton Dickinson FACS Aria) [16].

### H2B-GFP imaging

EW8 cells stably expressing H2B-GFP were generated using LentiBrite Histone H2B-GFP lentivirus (Millipore, #17-10229) [38]. Cells were imaged using an EVOS M5000 fluorescence microscope (ThermoFisher Scientific). Following drug treatment, metaphase and anaphase cells were manually identified and counted. A minimum of 200 cells were analyzed per replicate, and reviewers were blinded to treatment conditions.

### Apoptosis assays

The tetramethylrhodamine (TMRM) assay (ThermoFisher Scientific, T668) for mitochondrial membrane potential and the Annexin V assay (ThermoFisher Scientific, A13201) were performed according to the manufacturer’s instructions.

### EdU labeling and detection

Detection of DNA replication was performed in triplicate using a Click-iT EdU-488 kit (ThermoFisher Scientific) [13]. Briefly, cells were labeled with 10 mM EdU for one hour and then harvested using trypsin and fixed using the Click-iT fixative buffer. Cells were then washed by centrifugation and resuspended in Click-iT saponin-based permeabilization and wash reagent. Click-iT Plus reaction cocktail was added to the cell suspension for thirty minutes, after which the cells were washed by centrifugation. Flow cytometry was performed on a Becton Dickinson LSR II flow cytometer.

### γH2AX flow cytometry

Cells (3 × 10^5^ cells/well) were plated in a 6-well plate and allowed to adhere overnight. The cells were then treated with drugs or a vehicle, as described. Cells were labeled with EdU-488 for 2 h using the Click-iT EdU-488 kit for flow cytometry (ThermoFisher Scientific). Flow cytometry for γH2AX and EdU-488 was then performed as previously described [24].

### Puromycin labeling

Protein synthesis was assessed using puromycin labeling (SUnSET technique), as described [24, 35, 39, 40]. For labeling of newly synthesized proteins, puromycin (2 μg/mL) was added to cells at a 1:400 dilution. The cells were then incubated with the puromycin for one hour before the cell lysates were collected. Protein loading for the immunoblots was normalized using cell number.

### O-propargyl-puromycin labeling

Protein synthesis was assessed using O-propargyl-puromycin Click-iT labeling according to the manufacturer’s instructions (ThermoFisher Scientific) [41, 42]. Flow cytometry was performed using a Becton Dickinson LSR II instrument.

### Doxycycline-inducible shRRM1 and shCHK1

Sherwood UltramiR Lentiviral Inducible shRNA plasmids targeting shRRM1 and shCHK1 were obtained from Transomic Technologies (Huntsville, AL). Lentivirus was produced by transfecting HEK-293T cells with the shRNA plasmid and packaging plasmids (psPAX2 and pMD2.G) according to the FuGENE 6 (Roche) protocol. For the lentiviral transduction, Ewing sarcoma cells were incubated with 2 mL of virus and 6 mg/mL of polybrene (Sigma-Aldrich, TR-1003) for 12–16 h. Cells were selected in 1 μg/mL puromycin 48 h after transduction.

### Caspase-3/7 activation assay

Caspase-3/7 activation was measured using the Caspase-Glo 3/7 Luminescence assay (Promega, G8090), according to the manufacturer’s instructions.

### Reverse phase protein array (RPPA)

RPPA analysis of cell lines was performed by the RPPA Core Facility at the MD Anderson Cancer Center. Cells were provided to the core facility as frozen pellets, and the protein extraction, data normalization, and analysis were performed according to facility protocols (https://www.mdanderson.org/research/research-resources/core-facilities/functional-proteomics-rppa-core/education-and-references.html) (RRID:SCR_016649).

### Xenograft

The tumor samples used in this study were previously collected in an earlier study [24]. Briefly, approximately 1.0 × 10^6^ EW8 or TC71 cells were mixed with 30% matrigel and injected subcutaneously into the flanks of 6-week-old, female NCr mice. After tumors were palpable (~100–200 mm^3^), the mice were randomized and treated with vehicle, gemcitabine (150 mg/kg, intraperitoneal, day 1), prexasertib (10 mg/kg, subcutaneous, twice daily, day 1), or drug combination. On day 2, twenty-four h after gemcitabine administration, the mice were sacrificed, and the tumors were collected for analysis, without blinding, by immunoblotting. Two mice were treated in each cohort, and a power calculation was not performed. No animals were excluded from analysis. The Institutional Animal Care and Usage Committee at the University of Iowa approved the animal experiments, and the studies were conducted in adherence with the NIH Guide for the Care and Use of Laboratory Animals.

### Statistical analysis

Statistical analyses were conducted using GraphPad Prism 10.1.0 (RRID: SCR_002798). Data were presented as means with individual replicates shown. Single comparisons were analyzed by unpaired 2-tailed *t* test. Multiple comparisons were analyzed by 1-way ANOVA with Tukey’s post hoc multiple comparisons test. *P* values of less than or equal to 0.05 were considered significant.

### Study approval

The Institutional Animal Care and Usage Committee at the University of Iowa approved the animal experiment, and the studies were conducted in adherence with the NIH Guide for the Care and Use of Laboratory Animals.

## Results

### CHK1 inhibition induces DNA damage and apoptosis in S-phase Ewing sarcoma cells

**Fig. 1:**
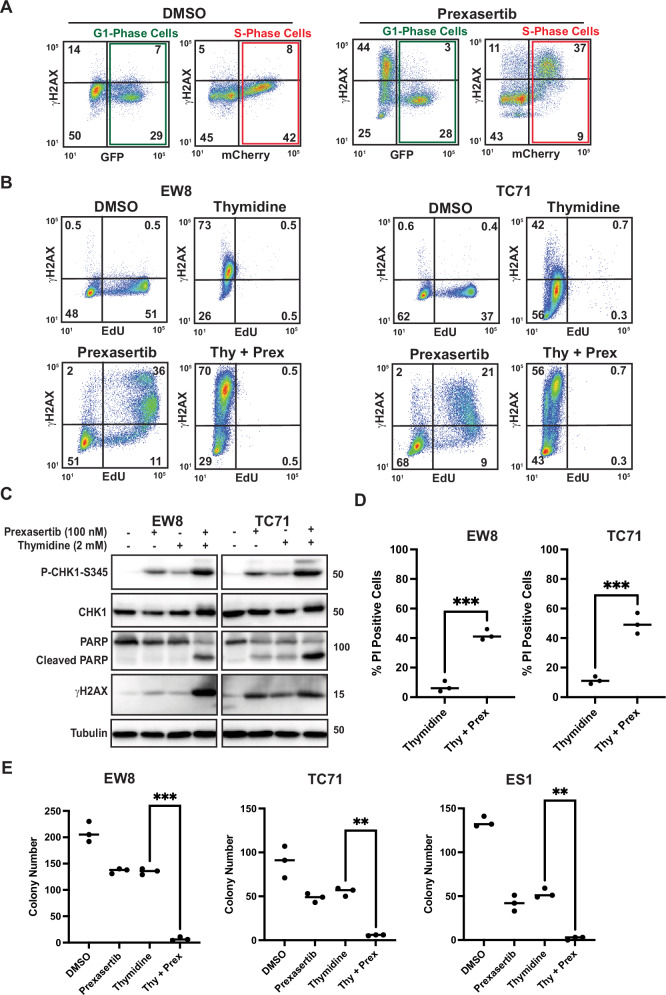
CHK1 inhibition induces DNA damage and cell death in S-phase–arrested Ewing sarcoma cells. **A** EW8 cells expressing PIP-FUCCI cell cycle markers were treated with prexasertib for 4 h and then analyzed by flow cytometry for γH2AX, G1 phase (GFP), and S phase (mCherry). **B** EW8 and TC71 cells were treated with DMSO, thymidine (h 0–22), prexasertib (h 18–22), or thymidine (h 0–22) with prexasertib (h 18–22) and then were labeled with EdU to mark actively replicating cells. Flow cytometry was performed for EdU incorporation and γH2AX expression. **C** Cells were treated with drugs as in (**B**) and then cellular lysates were collected for immunoblotting. **D** EW8 and TC71 cells were treated with thymidine (h 0–22) or thymidine (h 0–22) with prexasertib (h 18–22) and then the cells were stained with propidium iodide to identify dead cells. *n* = 3 replicates per condition. Data are representative of three independent experiments. **E** Cells were treated as in (**B**) and then the drugs were washed out and cells were allowed to recover and grow for 14 days in a colony formation assay. *n* = 3 replicates per condition. Data are representative of two independent experiments. Single comparisons (**D**) were analyzed by unpaired 2-tailed *t* test. Multiple comparisons (**E**) were analyzed by 1-way ANOVA with Tukey’s post hoc multiple comparisons test, with selected comparisons shown. *P* values of less than or equal to 0.05 were considered significant. **P < 0.01; ***P < 0.001.

The ATR-CHK1-WEE1 signaling pathway orchestrates the cellular response to DNA damage and replication stress, regulating key checkpoints across multiple phases of the cell cycle [22, 43, 44]. To determine the specific cell cycle phase in which ATR-CHK1-WEE1 inhibitors induce DNA damage, we treated EW8 Ewing sarcoma cells expressing the PIP-FUCCI fluorescent reporter system, which distinguishes the different phases of the cell cycle using the regulated expression of GFP- and mCherry-tagged protein fragments, with a CHK1 inhibitor (prexasertib) for four h [37]. Flow cytometry was then used to quantify γH2AX expression, a marker of DNA damage, alongside the fluorescent cell cycle indicators [45]. Figure 1A shows CHK1 inhibition-induced DNA damage specifically in S-phase cells, consistent with the observed synergy between ATR-CHK1-WEE1 inhibitors and DNA replication-targeting agents [13, 14, 24, 46]. We then used thymidine, a reversible inhibitor of the RRM1 subunit of RNR, to arrest cells in S-phase and determine whether active DNA replication is required for ATR-CHK1-WEE1 inhibitor toxicity. EW8 and TC71 cells were treated with DMSO, thymidine (2 mM for 22 h), prexasertib (100 nM for 4 h), or thymidine (h 0–22) with prexasertib (h 18–22). Cells were also labeled with 5-ethynyl-2’-deoxyuridine (EdU), a synthetic nucleoside used to detect newly synthesized DNA, and flow cytometry was performed for EdU and γH2AX (Fig. 1B) [47]. In the absence of thymidine, CHK1 inhibition induced γH2AX in EdU-positive cells, confirming DNA damage during active replication, consistent with the PIP-FUCCI results. However, significant DNA damage was also observed in EdU-negative, thymidine-arrested cells following CHK1 inhibition, demonstrating that ongoing DNA synthesis is not required to sensitize S-phase-arrested cells to inhibition of CHK1. CHK1 inhibition in thymidine-arrested cells also increased levels of cleaved PARP, a marker of apoptosis (Fig. 1C). To assess whether the combined thymidine (h 0–22) and prexasertib (h 18–22) treatment was sufficient to induce cell death, we stained cells with propidium iodide (PI), a fluorescent dye that marks dead or membrane-compromised cells. As shown in Fig. 1D, dual drug treatment significantly increased the percentage of PI-positive cells. Finally, to assess long-term effects on cell viability, Ewing sarcoma cell lines were treated with thymidine (h 0–22), prexasertib (h 18–22), or the combination. Drugs were then removed, and cells were cultured for an additional 14 days in a colony formation assay. Notably, even a 4-hour exposure to prexasertib, in combination with thymidine, was sufficient to markedly reduce colony-forming ability (Fig. 1E).

### CHK1 inhibition induces caspase-dependent apoptosis in S-phase Ewing sarcoma cells

**Fig. 2:**
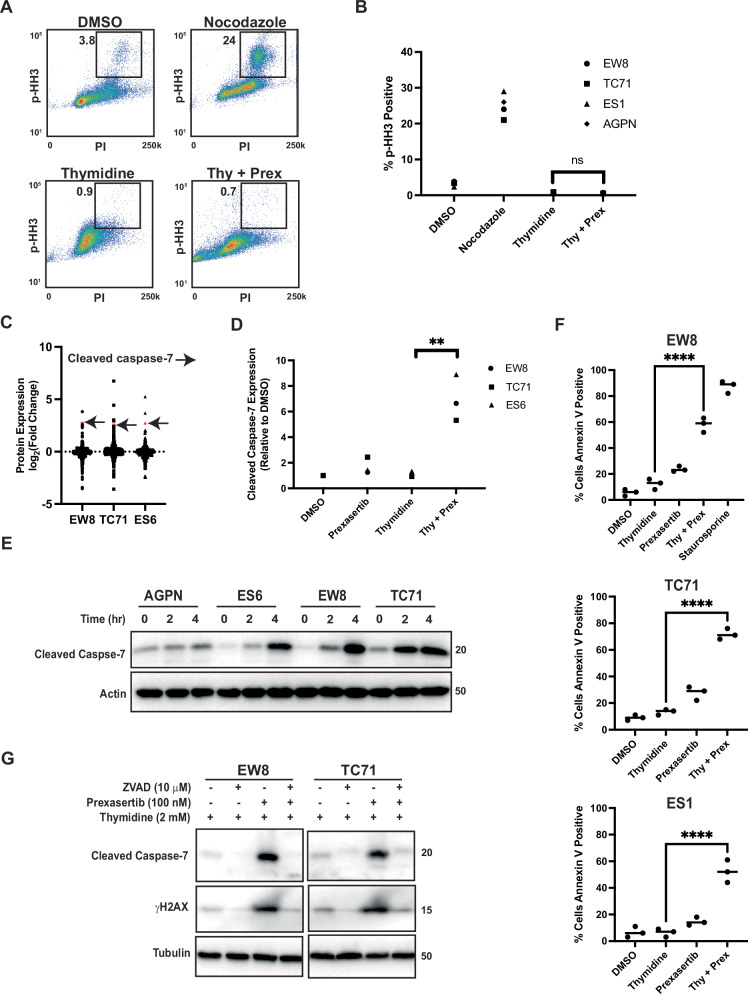
CHK1 inhibition triggers rapid caspase-dependent apoptosis in S-phase–arrested Ewing sarcoma cells without widespread mitotic entry. **A** Representative flow cytometry plots showing phospho-Histone H3 (p-HH3) staining in EW8 cells treated with DMSO, thymidine (h 0–22), thymidine (h 0–22) with prexasertib (h 18–22), or nocodazole (100 ng/mL for 4 h; positive control for mitotic arrest). **B** Quantification of p-HH3 staining across additional Ewing sarcoma cell lines treated as in (**A**). **C** Comparison of protein expression (RPPA) in Ewing sarcoma cells lines treated with thymidine (h 0–22) and prexasertib (h 18–22) versus thymidine (h 0–22) alone. Arrow indicates cleaved caspase-7. *n* = 1 replicate per condition per cell line. **D** Cleaved caspase-7 expression (RPPA) in Ewing sarcoma cells treated with DMSO, thymidine (h 0–22), prexasertib (h 18–22), or thymidine (h 0–22) with prexasertib (h 18–22). **E** Cells were treated with thymidine (h 0–22) and then prexasertib was added for 4 h (h 18–22). Protein lysates were collected at 0, 2, and 4 h after the addition of prexasertib. **F** Cells were treated with drugs as in (**D**) and then collected for flow cytometry for quantification of Annexin V positivity. Staurosporine was included as a positive control as a drug that induces apoptosis. *n* = 3 replicates per condition. Data are representative of three independent experiments. **G** EW8 and TC71cells were treated as in (**D**), with or without the pan-caspase inhibitor Z-VAD-FMK (h 18–22), and then lysates were collected for immunoblotting. Multiple comparisons (**B, D, F**) were analyzed by 1-way ANOVA with Tukey’s post hoc multiple comparisons test, with selected comparisons shown. *P* values of less than or equal to 0.05 were considered significant. ****P < 0.0001.

Inhibition of CHK1 in cells experiencing DNA replication stress can drive premature mitotic entry, through deregulation of CDC25 and CDK1 activity, even when DNA replication is incomplete [29–31]. Unexpectedly, treatment of Ewing sarcoma cells with thymidine and prexasertib did not increase phosphorylation of Histone H3 (p-HH3), a canonical marker of mitotic entry (Fig. 2A, B) [31]. To further evaluate mitotic progression, we used cells expressing H2B-GFP to directly visualize DNA and mitotic events [38]. Consistent with the Histone H3 findings, prexasertib treatment did not increase the number of mitotic cells (Supplementary Fig. 1A, B). Likewise, prexasertib did not increase phosphorylation of MPM2, a marker of mitosis, and drug-induced toxicity was not rescued by nocodazole, which arrests cells in early prometaphase and blocks mitotic progression (Supplementary Fig. 1C, D). The absence of cells entering mitosis raised the question of how prexasertib was driving toxicity. To identify proteomic changes induced by CHK1 inhibition in thymidine-arrested cells, we performed reverse phase protein array (RPPA) analysis on three Ewing sarcoma cell lines (Supplementary Table 1). Notably, cleaved caspase-7, a hallmark of apoptosis, was among the most highly upregulated proteins following prexasertib treatment (Fig. 2C, D) and was detectable as early as two h after drug exposure (Fig. 2E). Prexasertib treatment of thymidine-arrested cells also led to a reduction in mitochondrial membrane potential and an increase in Annexin V binding to exposed phosphatidylserine in the outer leaflet of the membrane, both of which are markers of apoptosis (Fig. 2F and Supplementary Fig. 2) [48]. Importantly, caspase-7 cleavage, assessed by both immunoblotting and a luminescence-based assay, and DNA damage were abrogated by co-treatment of the cell lines with the pan-caspase inhibitor Z-VAD-FMK, confirming the role of caspase activation in mediating these effects (Fig. 2G and Supplementary Fig. 3). Similar results were obtained using a double thymidine block to synchronize cells at the G1/S boundary, whereas a single thymidine block arrests cells more broadly across S-phase (Supplementary Fig. 4).

### Caspase inhibition rescues drug toxicity

**Fig. 3:**
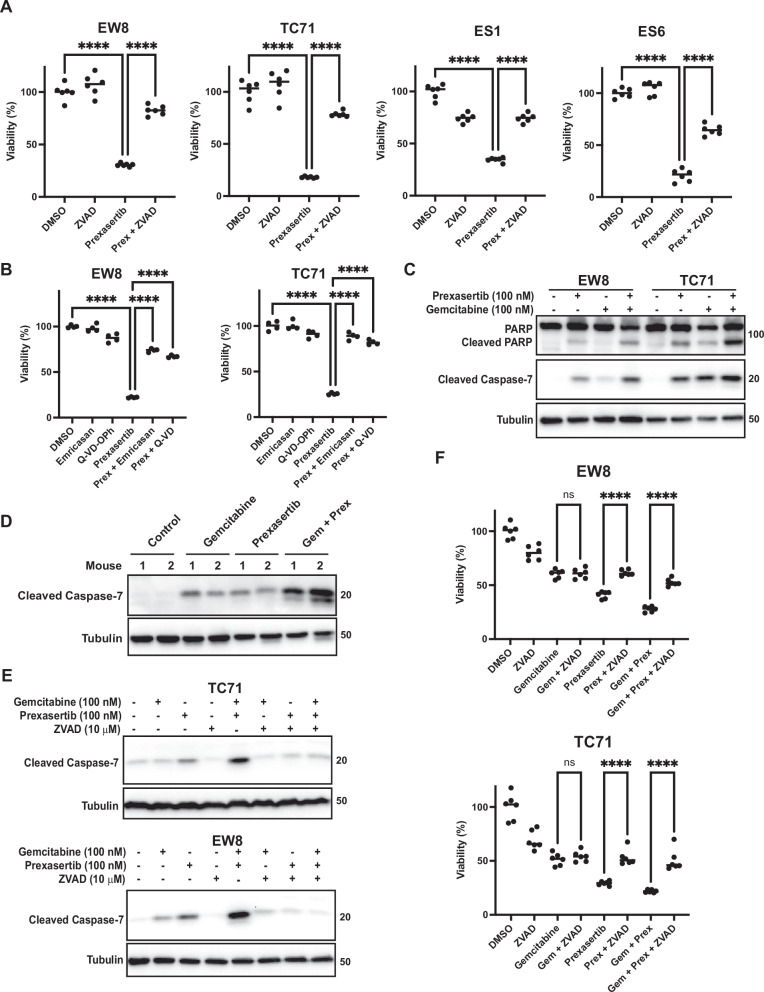
Caspase inhibition rescues the cytotoxic effects of combined replication stress and CHK1 inhibition in Ewing sarcoma cells. **A** EW8 and TC71 cells were synchronized in S-phase using thymidine treatment for 18 h. While maintaining thymidine treatment, cells were subsequently treated for 24 h with DMSO, prexasertib (100 nM), or prexasertib (100 nM) with Z-VAD-FMK (10 μM). Cell viability was then quantified using Cell-Titer-Glo. *n* = 6 replicates per condition. The data are representative of two independent experiments. **B** EW8 and TC71 cells were treated as in (**A**) except that two additional pan-caspase inhibitors (emricasan and Q-VD-OPh) were substituted for Z-VAD-FMK. **C** EW8 and TC71 cells were treated with gemcitabine, prexasertib, or the drug combination for 24 h. **D** Mice with TC71 xenograft tumors were treated with gemcitabine (150 mg/kg, intraperitoneal, day 1) and prexasertib (10 mg/kg, subcutaneous, twice daily, day 1) and then tumors were harvested 24 h after gemcitabine administration. **E** Ewing sarcoma cells were treated with gemcitabine and prexasertib, with or without Z-VAD-FMK, for 24 h. **F** Cells were treated as in (**E**) and then cell viability was quantified using Cell-Titer-Glo. *n* = 6 replicates per condition. Data are representative of two independent experiments. Multiple comparisons (**A, B, F**) were analyzed by 1-way ANOVA with Tukey’s post hoc multiple comparisons test, with selected comparisons shown. *P* values of less than or equal to 0.05 were considered significant. ****P < 0.0001.

To assess the impact of caspase inhibition on cell viability, we extended the duration of CHK1 inhibitor treatment from 4 h to 24 h. Ewing sarcoma cells were first treated with thymidine for 18 h to arrest the cells in S-phase, followed by a 24-h treatment with prexasertib in the presence or absence of the pan-caspase inhibitor Z-VAD-FMK. Cell viability was subsequently quantified, and as shown in Fig. 3A and Supplementary Fig. 5A, Z-VAD-FMK significantly rescued Ewing sarcoma cell viability under this more prolonged treatment condition. In contrast, several non-Ewing sarcoma cell lines were more resistant to the drug treatment and expression of the EWS::FLI1 oncogene in the osteosarcoma cell line U2OS increased sensitivity to the combination therapy (Supplementary Fig. 5B, C). Figure 3B and Supplementary Fig. 6A demonstrate that two additional pan-caspase inhibitors, emricasan and Q-VD-OPh, also significantly reduced drug-induced toxicity [49]. Next, to model a clinically relevant combination therapy, we replaced thymidine with gemcitabine, an FDA-approved ribonucleotide reductase (RNR) inhibitor. Ewing sarcoma cells were treated with gemcitabine, prexasertib, or the combination of both agents. As shown in Fig. 3C, this drug combination also increased the cleavage of caspase-7 and PARP. In previously published work, we treated mice bearing Ewing sarcoma xenograft tumors (TC71) with gemcitabine, prexasertib, or the combination, and collected tumors 24 h after drug administration [24]. Analysis of these stored tumor samples showed that the combination treatment increased cleavage of caspase-7, consistent with results obtained in vitro (Fig. 3D). Co-treatment with Z-VAD-FMK reduced caspase-7 cleavage (Fig. 3E) and enhanced cell viability (Fig. 3F) in response to the gemcitabine-prexasertib combination, indicating that caspase-dependent apoptosis contributes to the cytotoxic effects of this treatment. Inhibition of CHK1 also enhanced apoptosis in response to low-dose gemcitabine (as low as 1 nM) (Supplementary Fig. 6B). Additionally, hydroxyurea, an inhibitor of the RRM2 subunit of RNR, induced apoptosis when combined with prexasertib (Supplementary Fig. 6C).

### Loss of CHK1, using a CRISPR/Cas9-mediated CHK1 knockout-rescue model, induces apoptosis in S-phase Ewing sarcoma cells

**Fig. 4:**
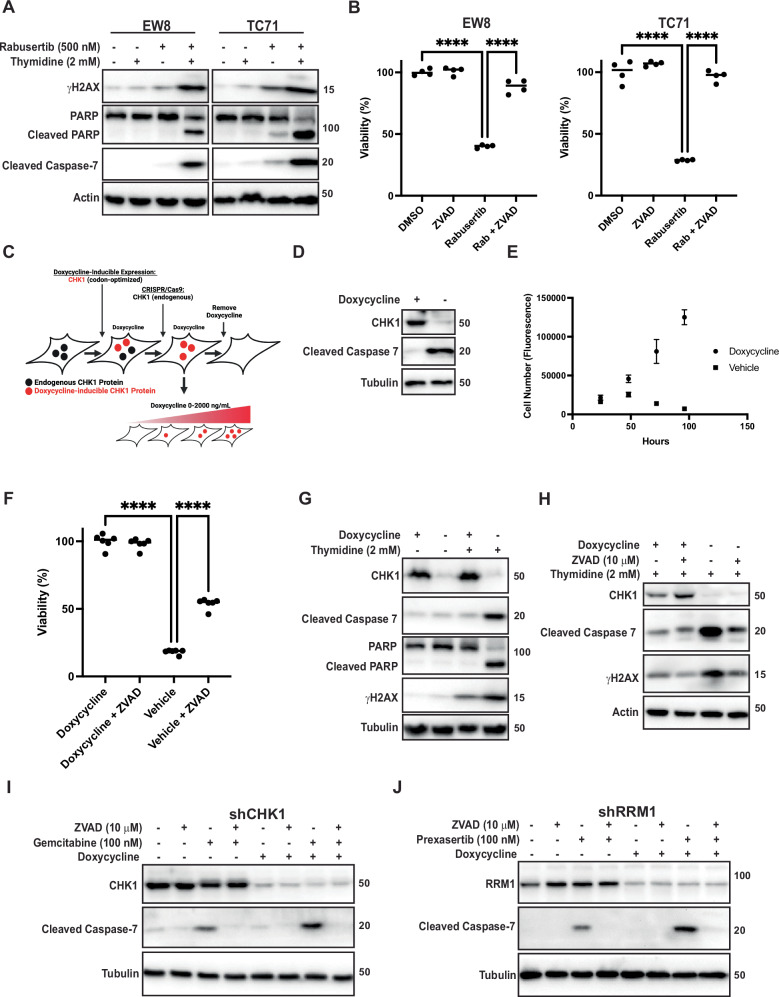
Pharmacologic and genetic suppression of CHK1 induces caspase-dependent apoptosis in Ewing sarcoma cells. **A** EW8 and TC71 cells were treated with DMSO, thymidine (h 0–22), rabusertib (h 18–22), or thymidine (h 0–22) with rabusertib (h 18–22). **B** Cells were treated with DMSO, thymidine (h 0–42), rabusertib (h 18–42), thymidine (h 0–42) with prexasertib (h 18–42), or thymidine and prexasertib with Z-VAD-FMK (h 18–42). Cell viability was then quantified using Cell-Titer-Glo. *n* = 4 replicates per condition. Data are representative of two independent experiments. **C** Schematic illustrating the approach used to knockout the endogenous CHK1 gene in Ewing sarcoma cells that express a doxycycline-inducible CHK1 transgene that is codon optimized and resistant to targeting by CRISPR/Cas9. **D** TO-CHK1-KO cells were cultured in the presence or absence of doxycycline for 24 h. **E** TO-CHK1-KO cells were cultured in the presence or absence of doxycycline, and cell number was quantified at multiple time points following doxycycline withdrawal. **F** TO-CHK-KO cells were treated as in (**D**), with or without Z-VAD-FMK, and then cell viability was then quantified using Cell-Titer-Glo. *n* = 6 replicates per condition. Data are representative of three independent experiments. **G** TO-CHK1-KO cells treated with thymidine for 18 h and then doxycycline was removed for 24 h. **H** Cells were treated as in (**G**) except that Z-VAD-FMK was added when the doxycycline was removed. **I** EW8 cells expressing a doxycycline-inducible shRNA targeting CHK1 were treated with doxycycline for 24 h and then gemcitabine, with or without Z-VAD-FMK, was added for 4 h. **J** EW8 cells expressing a doxycycline-inducible shRNA targeting RRM1 were treated with doxycycline for 24 h, and then prexasertib, with or without Z-VAD-FMK, was added for 4 h. Multiple comparisons (**B, F**) were analyzed by 1-way ANOVA with Tukey’s post hoc multiple comparisons test, with selected comparisons shown. *P* values of less than or equal to 0.05 were considered significant. ****, *P* < 0.0001.

Off-target effects are a well-recognized limitation of many kinase inhibitors, including those targeting CHK1 [50]. For example, prexasertib inhibits not only CHK1 but also CHK2 and RSK1 [51]. In contrast, rabusertib has been characterized as a more selective CHK1 inhibitor [50]. As shown in Fig. 4A, B, treatment with rabusertib induces apoptosis and reduces the viability of thymidine-arrested Ewing sarcoma cells, recapitulating the effects observed with prexasertib. To further investigate the specific role of CHK1 in regulating apoptosis, we employed a genetic approach [16]. We generated cell lines in which the endogenous CHK1 gene was knocked out using CRISPR/Cas9, while an exogenous, doxycycline-inducible, Cas9-resistant CHK1 transgene was stably expressed—referred to as TO-CHK1-KO cells (Fig. 4C). Consistent with CHK1 being an essential gene, doxycycline withdrawal led to CHK1 depletion, reduced cell viability and proliferation (Fig. 4D, E). This effect was significantly rescued by the pan-caspase inhibitor Z-VAD-FMK (Fig. 4F). Similarly, thymidine arrest followed by doxycycline withdrawal, leading to CHK1 depletion, induced apoptosis, which was also rescued by Z-VAD-FMK (Fig. 4G, H). Finally, in previously published work, we used doxycycline-inducible shRNAs to partially deplete CHK1 and RRM2 and sensitize cells to replication stress-inducing agents [13]. CHK1 knockdown increased gemcitabine-induced caspase-7 cleavage, while RRM1 knockdown enhanced prexasertib-induced caspase-7 cleavage, and both effects were abrogated by Z-VAD-FMK (Fig. 4I, J).

### Inhibition of CHK1 activates CDK1 and induces apoptosis

**Fig. 5:**
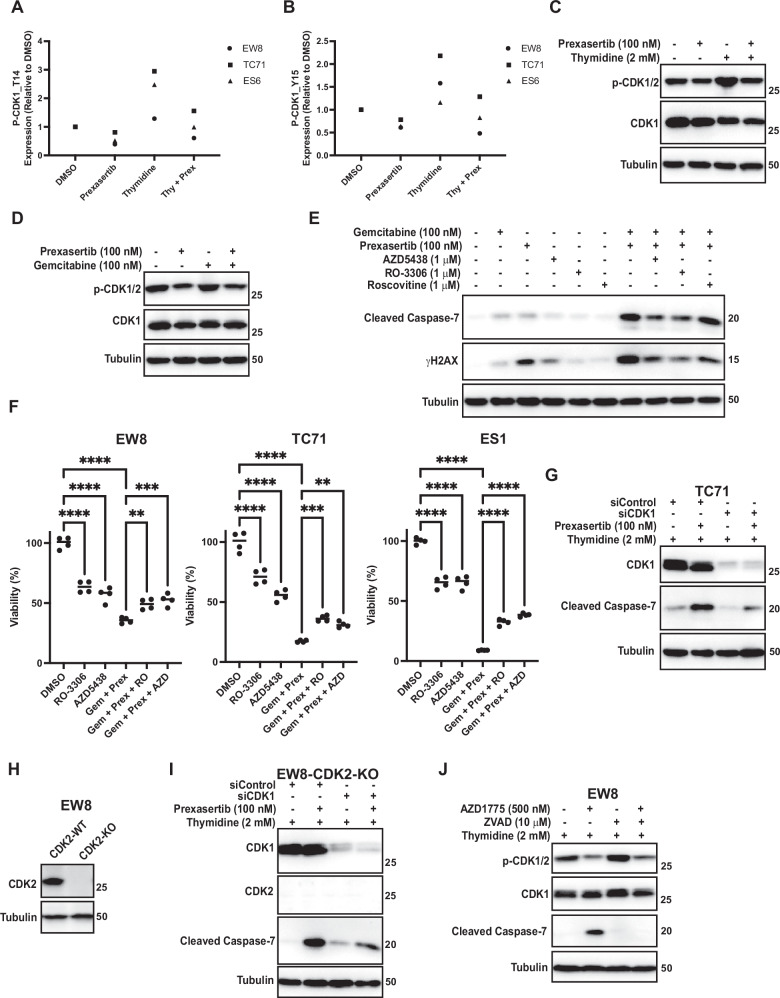
CDK1 activation is required for apoptosis induced by replication stress and ATR–CHK1–WEE1 pathway inhibition. **A, B** P-CDK1_T14 and P-CDK1_Y15 (RPPA) expression in Ewing sarcoma cells treated with DMSO, thymidine (h 0–22), prexasertib (h 18–22), or thymidine (h 0–22) with prexasertib (h 18–22). **C** EW8 cells were treated as in (**A**) and then lysates were collected for immunoblotting. **D** EW8 cells were treated with gemcitabine, prexasertib, or the drug combination for 24 h. **E** EW8 cells were treated with gemcitabine and prexasertib in combination with CDK1 (RO-3306 and AZD5438) and CDK2 inhibitors (roscovitine) for 24 h. **F** Ewing sarcoma cells were treated as in (**E**) and then cell viability was then quantified using Cell-Titer-Glo. *n* = 4 replicates per condition. Data are representative of three independent experiments. **G** TC71 cells were treated with siCDK1 (h 0–24), or siControl, followed by thymidine (h 24–46) and then prexasertib (h 42–46). **H, I** EW8 cells with CDK2-WT or CDK2-KO were treated as in (**G**) and then lysates were collected for immunoblotting. **J** EW8 cells were treated with thymidine (h 0–22) followed by AZD1775 (h 18–22). Multiple comparisons (**F**) were analyzed by 1-way ANOVA with Tukey’s post hoc multiple comparisons test, with selected comparisons shown. *P* values of less than or equal to 0.05 were considered significant. **P < 0.01; ***P < 0.001; ****, *P* < 0.0001.

Analysis of the reverse phase protein array data identified that CHK1 inhibition in thymidine-arrested Ewing sarcoma cells reduced the phosphorylation of CDK1 at T14 and Y15 (Fig. 5A, B). Under normal conditions, CDC25 activates CDK1 by dephosphorylation, promoting mitotic entry. In contrast, under conditions of replication stress, ATR-CHK1 signaling inhibits CDC25 to prevent premature mitosis, while WEE1 suppresses CDK1 directly via phosphorylation [44]. However, CDK1 not only governs mitotic entry but also plays a role in regulating apoptosis [52–55]. As shown in Fig. 5C, D, thymidine or gemcitabine treatment increased CDK1 phosphorylation, an effect reversed by prexasertib. Importantly, inhibition of CDK1 using RO-3306 or AZD5438 significantly reduced caspase-7 cleavage and γH2AX phosphorylation induced by gemcitabine and prexasertib (Fig. 5E). Similar results were obtained with a CDC25 inhibitor (NSC663284) and an additional CDK1 inhibitor, dinaciclib (Supplementary Fig. 7A, B) [56]. In contrast, roscovitine, a more CDK2-selective inhibitor, did not block caspase-7 cleavage, though it did suppress γH2AX phosphorylation, indicating a distinct role for CDK1 in apoptosis. While CDK1 is an essential protein and its inhibition alone is cytotoxic in Ewing sarcoma cells, CDK1 inhibitors (RO-3306 or AZD5438) nonetheless attenuated the combined cytotoxicity of gemcitabine and prexasertib in viability assays (Fig. 5F). Additionally, siRNA-mediated CDK1 knockdown reduced caspase-7 cleavage (Fig. 5G). In contrast, CDK2, a non-essential kinase, was knocked out using CRISPR/Cas9 (CDK2-KO), and its loss did not affect caspase-7 cleavage (Fig. 5H, I). However, siRNA knockdown of CDK1 in CDK2-KO cells suppressed caspase-7 cleavage, further underscoring the role of CDK1 in drug-induced apoptosis (Fig. 5I). Inhibition of ATR, which functions upstream of CHK1, also induced apoptosis in thymidine-arrested cells (Supplementary Fig. 8A) [22]. Furthermore, combined treatment with thymidine (0–22 h) and the WEE1 inhibitor AZD1775 (18–22 h) increased caspase-7 cleavage (Fig. 5J) [57]. Inhibition of PKMYT1, a kinase that specifically phosphorylates and inhibits CDK1, using RP-6306 also enhanced caspase-7 cleavage in thymidine-treated cells (Supplementary Fig. 8B) [58]. This effect was blocked by Z-VAD-FMK, confirming caspase dependence. Additionally, inhibition of anti-apoptotic Bcl-2 proteins with navitoclax or venetoclax further enhanced apoptosis (Supplementary Fig. 8C, D). Finally, the reverse phase protein array data identified that, in addition to cleaved caspase-7, cleaved caspase-8 is significantly upregulated following prexasertib treatment in thymidine-arrested cells (Supplementary Fig. 9A). Cleaved caspase-8, an initiator caspase that regulates the extrinsic apoptotic pathway and activates the executioner caspases-3/7, was detectable as early as two h after drug exposure (Supplementary Fig. 9B). To confirm its functional role, we treated Ewing sarcoma cells with thymidine for 18 h to arrest cells in S-phase, followed by a 24-hour exposure to prexasertib in the presence or absence of Z-IETD-FMK, a selective caspase-8 inhibitor. Z-IETD-FMK significantly rescued drug-induced toxicity, although to a lesser extent than the pan-caspase inhibitor Z-VAD-FMK (Supplementary Fig. 9C, D). In contrast, inhibitors targeting caspase-2 (Z-VDVAD-FMK), caspase-9 (Z-LEHD-FMK), or caspase-10 (Z-AEVD-FMK), which are additional initiator caspases, did not attenuate prexasertib toxicity.

### Drug-induced inhibition of protein synthesis is independent of apoptosis

**Fig. 6:**
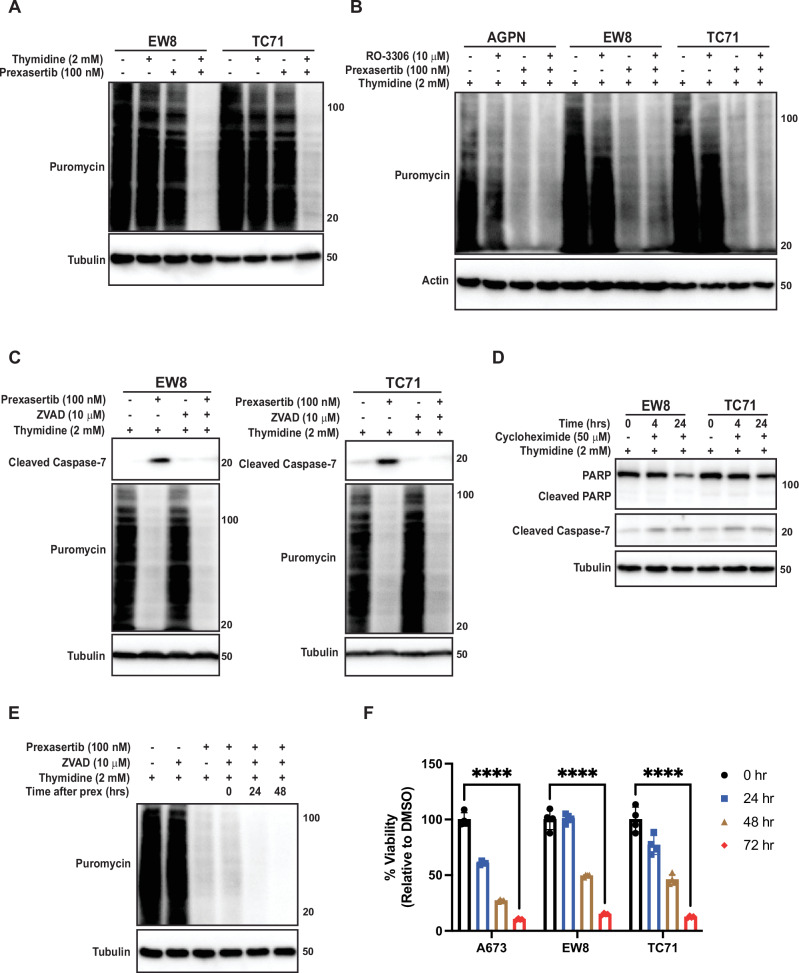
Drug-induced suppression of protein synthesis occurs independently of CDK1 activation and caspase-mediated apoptosis. **A** Ewing sarcoma cell lines were treated were treated with DMSO, thymidine (h 0–22), prexasertib (h 18–22), or thymidine (h 0–22) with prexasertib (h 18–22). Puromycin was added for the last hour of drug incubation to label newly synthesized proteins. Puromycin was then detected by immunoblotting. **B** Ewing sarcoma cells lines were treated as in (**A**) except that a CDK1 inhibitor, RO-3306, was added with prexasertib from h 18–22. **C** Ewing sarcoma cells lines were treated as in (**A**) except that a pan-caspase inhibitor, Z-VAD-FMK, was added with prexasertib from h 18–22. **D** Ewing sarcoma cells lines were treated DMSO, thymidine (h 0–22), cycloheximide (h 18–22) or thymidine (h 0–22) with cycloheximide (h 18–22). **E** EW8 cells were treated with DMSO, thymidine (0–22 h), prexasertib (18–22 h), or prexasertib with Z-VAD-FMK (18–22 h). Prexasertib was removed at hour 22, and cells were subsequently cultured in the presence of Z-VAD-FMK for an additional 48 h. **F** Ewing sarcoma cell lines were treated with cycloheximide (50 μM) and then cell viability was quantified at different time points using Cell-Titer-Glo. *n* = 4 replicates per condition. The data are representative of two independent experiments. Multiple comparisons were analyzed by 1-way ANOVA with Tukey’s post hoc multiple comparisons test, with selected comparisons shown. *P* values of less than or equal to 0.05 were considered significant. ****, *P* < 0.0001.

In previous studies, we identified that dual inhibition of ribonucleotide reductase (RNR) and CHK1 suppresses protein synthesis in Ewing sarcoma cell lines and xenograft models [24, 35]. Similarly, combining an ATR inhibitor with cisplatin was shown to inhibit protein synthesis in Ewing sarcoma tumors [19]. These findings led us to investigate whether apoptosis plays a regulatory role in protein synthesis. As shown in Fig. 6A and Supplementary Fig. 10, the combination of thymidine and prexasertib significantly reduced protein synthesis, as measured by puromycin incorporation into nascent proteins [39]. However, this suppression was not reversed by inhibition of CDK1 or caspases (Fig. 6B, C). Consistently, Z-VAD-FMK also failed to restore protein synthesis in cells treated with gemcitabine and prexasertib (Supplementary Fig. 11A). To further assess whether inhibition of protein synthesis alone is sufficient to induce apoptosis, thymidine-arrested cells were treated with the protein synthesis inhibitor cycloheximide for four h. Although cycloheximide effectively blocked protein synthesis, it did not induce apoptosis, even when treatment was extended to 24 h (Fig. 6D). We next treated Ewing sarcoma cells with DMSO, thymidine (0–22 h), prexasertib (18–22 h), or prexasertib with Z-VAD-FMK (18–22 h). After removing prexasertib at hour 22, cells were cultured in the continued presence of Z-VAD-FMK, to block apoptosis, for an additional 48 h. Puromycin labeling at multiple time points following prexasertib removal revealed that suppression of protein synthesis persisted for at least 48 h (Fig. 6E). Finally, as shown in Fig. 6F, cycloheximide-mediated inhibition of protein synthesis was sufficient to significantly reduce cell viability. Together, these results demonstrate that drug-induced inhibition of protein synthesis and apoptosis occur through distinct, independent mechanisms and that inhibition of protein synthesis, which causes a long-term reduction in cell growth, persists after drug removal.

### Inhibition of protein synthesis restricts cell cycle progression in Ewing sarcoma cells

**Fig. 7:**
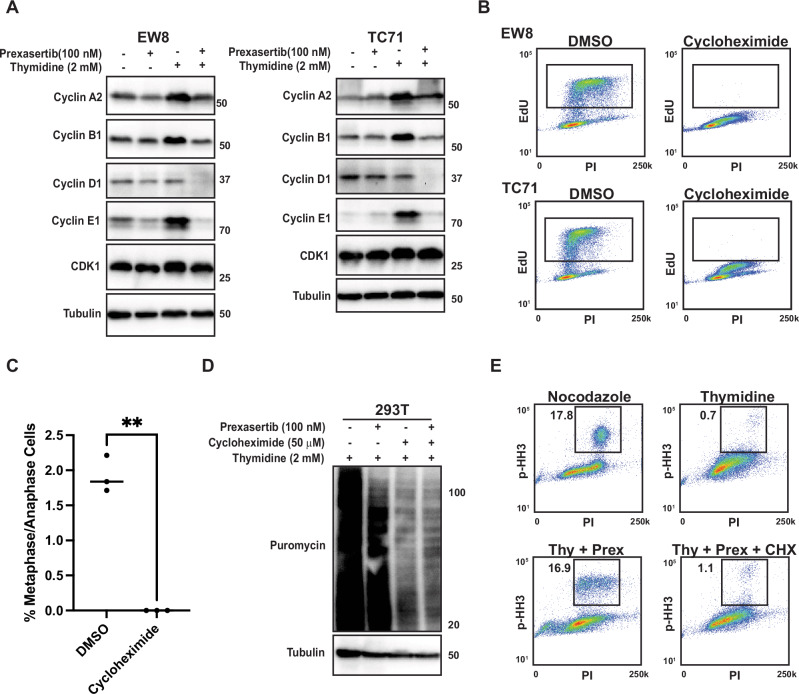
Inhibition of protein synthesis restricts cell-cycle progression and prevents mitotic entry. **A** Ewing sarcoma cell lines were treated were treated with DMSO, thymidine (h 0–22), prexasertib (h 18–22), or thymidine (h 0–22) with prexasertib (h 18–22). **B** EW8 and TC71 cells were treated with cycloheximide, an inhibitor of protein synthesis, for four h. Cells were then labeled with EdU to mark actively replicating cells, and cell cycle analysis was performed using flow cytometry. **C** EW8 cells expressing H2B-GFP were treated with thymidine (h 0–18) and then released from arrest in the presence or absence of cycloheximide. Metaphase and anaphase cells were manually counted. *n* = 3 replicates per condition with a minimum of 200 cells per replicate. Data were analyzed by unpaired 2-tailed *t* test with a *P* value of less than or equal to 0.05 considered significant. **D** HEK-293T cells were treated with thymidine (h 0–22), prexasertib (h 18–22), and thymidine (h 0–22) with prexasertib (h 18–22), with or without cycloheximide (h 18–22). **E** HEK-293T cells were treated with nocodazole (positive control) or drugs as described in (**D**). Flow cytometry for p-HH3 was performed to assess mitotic entry. **, *P* < 0.01.

Protein synthesis requirements vary throughout the cell cycle [59, 60]. Because cyclins are short-lived proteins that regulate CDK activity, we assessed their levels by immunoblotting following treatment with thymidine and prexasertib [61]. As shown in Fig. 7A, dual inhibition of RNR and CHK1 downregulated multiple cyclins, including cyclin B1, which regulates the G2-M transition [61]. We therefore asked whether drug-induced inhibition of translation might prevent mitotic entry, thereby directing Ewing sarcoma cells toward apoptosis rather than premature mitosis. To test this, cells were treated with cycloheximide to inhibit protein synthesis, followed by cell cycle analysis using EdU and propidium iodide. As shown in Fig. 7B, cycloheximide markedly reduced EdU incorporation, consistent with inhibition of DNA replication and S-phase progression. We next treated H2B-GFP-expressing cells with thymidine for 18 h to arrest them in S-phase, then released them into media with or without cycloheximide, and quantified mitotic entry by scoring metaphase and anaphase cells. Figure 7C demonstrates that cycloheximide significantly blocked S-phase progression into mitosis. We then extended these studies to HEK-293T cells, a (non-Ewing sarcoma) human embryonic kidney cell line, treated with thymidine and prexasertib. Unlike Ewing sarcoma cells, HEK-293T cells did not downregulate protein synthesis in response to the drug combination, although cycloheximide effectively blocked protein synthesis (Fig. 7D). We then treated HEK-293T cells with thymidine and prexasertib, with or without cycloheximide, and quantified mitotic entry using phosphorylation of histone H3 (p-HH3) (Fig. 7E). Notably, thymidine plus prexasertib increased p-HH3, but this effect was blocked by concurrent treatment with cycloheximide. Finally, Supplementary Fig. 11B shows that dual inhibition of WEE1 and CDC7 (DDK), previously shown to increase phosphorylation of histone H3 and induce premature mitotic entry in Ewing sarcoma cells, did not impair protein synthesis in these cells [26, 62]. However, cycloheximide blocked the increase in phosphorylation of histone H3 caused by this drug combination (Supplementary Fig. 11C). Together, these findings indicate that protein synthesis is a critical regulator of cell cycle progression and mitotic entry.

## Discussion

**Fig. 8:**
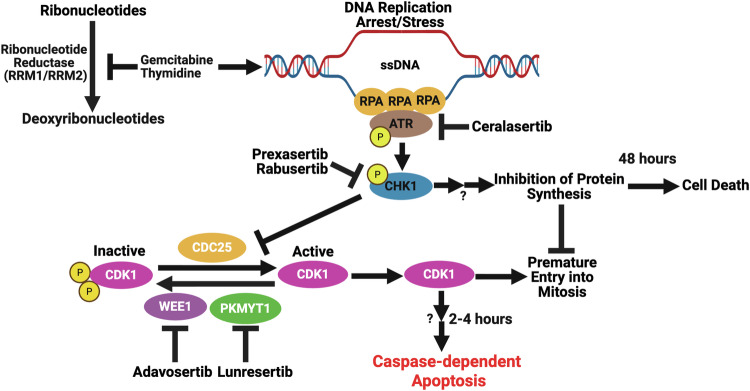
Dual targeting of ribonucleotide reductase and ATR-CHK1-WEE1 signaling induces CDK1-dependent apoptosis in Ewing sarcoma cells without mitotic entry. Ribonucleotide reductase inhibitors block deoxyribonucleotide synthesis, inducing DNA replication stress and S-phase arrest, which activates the ATR-CHK1-WEE1 DNA damage response pathway. Simultaneous inhibition of CHK1, or related kinases, leads to CDK1 activation. However, dual drug treatment also suppresses protein synthesis, preventing the accumulation of cyclins and other proteins required for cell-cycle progression and mitotic entry. As a result, activated CDK1 triggers rapid, caspase-dependent apoptosis within 2–4 h while cells remain in S phase, without premature mitotic entry. Inhibition of protein synthesis persists after drug withdrawal, contributing to long-term growth suppression in cells that evade apoptosis. The question marks in the schematic denote unresolved mechanistic steps.

In this study, we identified a novel mechanism by which ATR-CHK1-WEE1 pathway inhibition induces cell death in Ewing sarcoma cells experiencing DNA replication stress (Fig. 8). Our findings challenge the prevailing model that attributes the cytotoxic effects of these inhibitors primarily to forced mitotic entry and chromosomal instability [26, 28–33]. Instead, we demonstrate that S-phase-arrested Ewing sarcoma cells undergo rapid, CDK1-dependent, caspase-mediated apoptosis within 2–4 h of CHK1 inhibition, independent of widespread mitotic entry.

The rapid induction of apoptosis following CHK1 inhibition in S-phase-arrested cells represents a previously unrecognized mechanism of cell death in Ewing sarcoma. Unlike the conventional model, where ATR-CHK1-WEE1 inhibitors force premature mitotic entry leading to mitotic catastrophe, our data demonstrate that Ewing sarcoma cells can undergo apoptosis while remaining in S-phase. This finding is particularly significant given that we observed substantial DNA damage and cell death even in thymidine-arrested cells where active DNA replication was blocked, indicating that ongoing DNA synthesis is not required for sensitization to CHK1 inhibition. The absence of increased Histone H3 phosphorylation and direct visualization of mitotic events using H2B-GFP-expressing cells confirmed that the observed cell death occurs without widespread mitotic entry. This mechanism appears to be conserved across multiple Ewing sarcoma cell lines and is recapitulated both pharmacologically with multiple CHK1 inhibitors (prexasertib and rabusertib) and genetically using our CRISPR/Cas9-mediated CHK1 knockout-rescue model. Moreover, similar results were obtained using inhibitors of ATR, WEE1, and PKMYT1.

A central finding of our study is the identification of CDK1 as a critical mediator of apoptosis in this context. Our reverse-phase protein array analysis revealed that CHK1 inhibition leads to CDK1 activation, evidenced by reduced inhibitory phosphorylation at T14 and Y15 residues. Importantly, pharmacological inhibition of CDK1 with RO-3306 or AZD5438, as well as siRNA-mediated CDK1 knockdown, significantly reduced caspase-7 cleavage and DNA damage, demonstrating that CDK1 activation is necessary for the apoptotic response. The specificity of this effect was further validated by our observation that CDK2 knockout cells remained susceptible to drug-induced apoptosis, while subsequent CDK1 knockdown in these cells rescued the apoptotic phenotype. This finding underscores the unique role of CDK1, as opposed to other cyclin-dependent kinases, in mediating this form of cell death. While CDK2 has been implicated in regulating RRM2 expression during DNA replication, our findings underscore a more central role for CDK1 in mediating apoptosis in S-phase arrested cells [13, 63]. The involvement of WEE1 and PKMYT1 inhibition in enhancing caspase activation further supports the model that relief of CDK1 inhibitory phosphorylation is a key determinant of the apoptotic response.

Although CDK1 is classically recognized for its role in driving mitotic entry, accumulating evidence indicates that CDK1 can also promote apoptosis under certain cellular conditions [52–54, 64–66]. For example, in cells undergoing prolonged mitotic arrest, aberrant or unscheduled CDK1 activation can trigger apoptosis through mechanisms such as degradation of anti-apoptotic proteins and caspase activation [65–68]. In our study, we also identified a role for caspase-8, previously implicated in Ewing sarcoma biology, in regulating apoptosis in response to drug treatment [69–73]. However, inhibition of caspase-8 only partially rescued drug-induced cytotoxicity, suggesting that caspase-8 contributes to, but is not solely responsible for, cell death in this context. Additionally, we show that BCL-2 inhibitors enhance apoptosis, supporting a role for the intrinsic (mitochondrial) apoptosis pathway. Together, these findings suggest that both intrinsic and extrinsic apoptosis pathways likely contribute to the observed phenotype. Future work will employ proteomic and screening approaches to further define the specific proteins and pathways regulated by CDK1 in Ewing sarcoma cells. Importantly, these findings highlight CDK1 as a potential therapeutic target, where strategies that exploit its pro-apoptotic functions could enhance treatment efficacy in Ewing sarcoma.

Our discovery that the dual targeting of DNA replication and ATR-CHK1-WEE1 signaling suppresses protein synthesis provides a mechanistic explanation for why aberrant CDK1 activation does not drive mitotic entry in Ewing sarcoma cells. Protein synthesis is essential for cell cycle progression, particularly for the accumulation of cyclins and other regulatory proteins required for mitosis [60, 74]. The drug-induced suppression of protein synthesis effectively prevents cells from progressing through the cell cycle checkpoints necessary for mitotic entry, despite CDK1 activation. Notably, we found that protein synthesis inhibition occurs independently of both CDK1 activation and caspase-mediated apoptosis, as neither CDK1 inhibitors nor pan-caspase inhibitors restored protein synthesis in drug-treated cells. This suggests that protein synthesis suppression represents a distinct and parallel response to replication stress that ultimately determines cell fate by preventing mitotic progression and channeling cells toward apoptosis. Additionally, in previously published work, we showed that the EWS::FLI1 oncoprotein contributes to the aberrant regulation of protein synthesis in Ewing sarcoma cells, potentially explaining the vulnerability of these tumors to this therapeutic approach [24]. However, we also note that drugs targeting DNA replication stress are reported to inhibit protein synthesis in other cancer types as well, suggesting the effect is not limited to EWS::FLI1 and that other oncogenes may also have a role [75, 76].

Our data support a model wherein the therapeutic efficacy of replication stress-targeting combinations involves two complementary mechanisms operating on different timescales. The rapid induction of CDK1-dependent, caspase-mediated apoptosis represents the primary mechanism of acute cytotoxicity, eliminating the most vulnerable cells within 2–4 h of treatment. However, our findings also indicate that cells surviving this initial apoptotic wave face a secondary challenge in the form of sustained inhibition of protein synthesis. This translational repression likely contributes to long-term growth inhibition and eventual cell death through distinct pathways, including impaired DNA repair, compromised stress responses, and inability to synthesize essential survival proteins. The dual nature of this response may explain the long-term efficacy observed in our clonogenic assays, where even brief drug exposures resulted in marked reductions in colony-forming ability that exceeded the immediate apoptotic response. Cells that initially resist apoptosis due to heterogeneous expression of pro- and anti-apoptotic factors, or differences in cell cycle positioning, remain compromised by persistent translational defects that ultimately prove lethal over more extended periods. This model suggests that therapeutic combinations targeting both replication stress and checkpoint signaling may be particularly effective because they simultaneously engage acute apoptotic mechanisms while establishing chronic growth-inhibitory conditions that prevent long-term survival and proliferation.

The identification of this CDK1- and caspase-dependent apoptotic mechanism has implications for the clinical development of replication stress-targeting therapies in Ewing sarcoma. Our findings suggest that the efficacy of ATR-CHK1-WEE1 inhibitors may not rely on their ability to force mitotic entry, but rather on their capacity to activate pro-apoptotic signaling pathways in S-phase-arrested cells. This mechanistic insight could inform biomarker development and the identification of additional drug combinations with activity toward Ewing sarcoma tumors. For example, CDK1 phosphorylation status, cleaved caspase-7, and depletion of short-lived proteins as a marker of translational repression could serve as pharmacodynamic biomarkers of drug activity in ongoing clinical trials testing ATR and CHK1 inhibitors. The demonstration that brief exposure (2–4 h) to the combination of ribonucleotide reductase and CHK1 inhibitors is sufficient to markedly reduce long-term clonogenic survival suggests that intermittent dosing strategies might be effective while potentially reducing toxicity. Furthermore, our validation of these mechanisms in xenograft tumor models using the clinically relevant combination of gemcitabine and prexasertib supports the translational potential of these findings. Understanding the molecular basis of this apoptotic response also provides insights into potential mechanisms of resistance. Because caspase activation is essential for this form of cell death, tumors with defective apoptotic machinery or upregulated anti-apoptotic signaling may evade these drug combinations. Notably, we found that agents targeting anti-apoptotic proteins, such as venetoclax and navitoclax, enhance apoptosis, highlighting a promising direction for further investigation. In addition, alterations in CDK1 regulation or disruptions in protein synthesis machinery could also confer resistance.

Several mechanistic limitations should be acknowledged. First, the precise molecular mechanisms linking CHK1 inhibition to suppression of protein synthesis remain to be defined. In prior work, we showed that inhibition of RNR and CHK1 activates the translational repressor 4E-BP1, thereby suppressing protein synthesis [24, 35]. This mechanism was not evaluated in the current study but represents an area for future investigation. However, activation of 4E-BP1 alone did not fully account for the observed reduction in protein synthesis, indicating that additional mechanisms contribute. For example, Jess et al. demonstrated that combined cisplatin and ATR inhibition suppresses protein synthesis in Ewing sarcoma cells through activation of the unfolded protein response (UPR) and phosphorylation of eIF2α [19]. Additionally, effects of SLFN11, which is upregulated in Ewing sarcoma tumors and regulates protein synthesis, on ribosome biogenesis or processivity could also be hypothesized to impact protein translation [5, 77–79]. Further defining the mechanisms that regulate protein synthesis in Ewing sarcoma, including how replication stress pathways interface with translational control, will be a focus of future studies. Second, although we demonstrate an essential role for CDK1 in mediating apoptosis, the downstream effectors that directly link CDK1 activity to caspase activation have not been fully characterized. Nonetheless, as noted above, CDK1 can promote apoptosis through multiple mechanisms, including degradation of anti-apoptotic proteins such as Bcl-2, Bcl-xL, and MCL1; activation of the pro-apoptotic protein BAD; and regulation of caspase activation, all of which represent areas for future investigation [65–68]. Moreover, understanding the involvement of anti-apoptotic proteins in modulating CDK1-driven cell death may enable rational combination strategies (e.g., with BH3 mimetics) to preempt resistance. Third, additional studies will be required to determine which cyclin or cyclins partner with CDK1 to drive apoptotic signaling.

In summary, our work reveals a previously unrecognized mechanism by which ATR-CHK1-WEE1 pathway inhibitors kill cancer cells under replication stress. The identification of CDK1-dependent, caspase-mediated apoptosis as a primary mechanism of cell death in S-phase-arrested Ewing sarcoma cells challenges existing models and provides new insights into the molecular basis of therapeutic vulnerability in this aggressive pediatric malignancy. The demonstration that protein synthesis suppression prevents mitotic entry while CDK1 activation drives apoptosis offers a mechanistic framework that could guide the development of more effective replication stress-based therapeutic strategies. These findings not only advance our understanding of how Ewing sarcoma cells respond to replication stress but also provide a foundation for optimizing combination therapies targeting DNA replication and checkpoint signaling pathways. As these therapeutic approaches continue to advance through clinical trials, our mechanistic insights may prove valuable for maximizing therapeutic efficacy while minimizing the development of resistance.

## Supplementary information

Supplemental Figure Legends

Supplemental Table 1

Supplemental Figures

## Data Availability

Unedited immunoblots are available in the Unedited blot images file. Data values for all graphs are provided in the supporting data values file.
