# Supplementary material for: DNA replication stress and translational repression converge to drive CDK1- and caspase-dependent apoptosis in Ewing sarcoma
Source: Oncogene. 2026 Jun 10;45(28):2823–39. doi: 10.1038/s41388-026-03845-2 (PMC13337492; doi:10.1038/s41388-026-03845-2)
Supplement: Supplementary file 1 — Supplemental Figure Legends [file 41388_2026_3845_MOESM1_ESM.docx]

**SUPPLEMENTAL** **FIGURE LEGENDS**

**Supplemental Figure 1.** (A) Representative fluorescence images of EW8 cells expressing eGFP-H2B in metaphase (yellow arrow) and anaphase (white arrow). (B) EW8 cells expressing eGFP-H2B were treated with DMSO, thymidine (2 mM, hours 0-22), or thymidine (2 mM, hours 0-22) with prexasertib (100 nM, hours 18–22) and then metaphase and anaphase cells were manually counted. n=3 replicates per condition with a minimum of 200 cells per replicate. Multiple comparisons were analyzed by 1-way ANOVA with Tukey’s post hoc multiple comparisons test. *P* values of less than or equal to 0.05 were considered significant. ***P < 0.001. (C) Ewing sarcoma cell lines were treated with DMSO, thymidine (2 mM, hours 0-22), thymidine (2 mM, hours 0-22) with prexasertib (100 nM, hours 18–22), or nocodazole (50 ng/mL, hours 0-22). Cell lysates were then collected for immunoblotting. Nocodazole was used as a positive control to arrest cells in mitosis and increase p-MPM2. (D) EW8 cells were treated with DMSO, thymidine (2 mM, hours 0-22), thymidine (2 mM, hours 0-22) with prexasertib (100 nM, hours 18–22), thymidine (2 mM, hours 0-22) with prexasertib (100 nM, hours 18–22) and the CDK1 inhibitor RO-3306 (1 μM, hours 18-22), or thymidine (2 mM, hours 0-22) with prexasertib (100 nM, hours 18–22) and nocodazole (50 ng/mL, hours 18-22).

**Supplemental Figure 2.** EW8 and TC71 cells were treated with DMSO, thymidine (2 mM, hours 0-22), thymidine (2 mM, hours 0-22) with prexasertib (100 nM, hours 18–22), or staurosporine (1 μM, hours 18-22; positive control for apoptosis) and then mitochondrial membrane potential was measured using tetramethylrhodamine.

**Supplemental Figure 3.** EW8 and TC71 cells were treated with thymidine (hours 0-42), prexasertib (hours 18-42), thymidine (hours 0-42) with prexasertib (hours 18-42), or thymidine and prexasertib with Z-VAD-FMK (hours 18-42). Caspase-3/7 activation was then measured using the Caspase-Glo 3/7 Luminescence assay. n=4 replicated per condition. Multiple comparisons were analyzed by 1-way ANOVA with Tukey’s post hoc multiple comparisons test. *P* values of less than or equal to 0.05 were considered significant. ****P < 0.0001.

**Supplemental Figure 4.** EW8 and TC71 cells were synchronized using a double thymidine block, followed by treatment with prexasertib (100 nM) for 4 hours, either alone or in combination with Z-VAD-FMK (10 μM). Cellular lysates were then collected for immunoblot analysis.

**Supplemental Figure 5.** (A-B) Ewing sarcoma (A673, SKNEP, and TTC466) and non-Ewing sarcoma (HT1080, RD, and U2OS) cell lines were synchronized in S-phase using thymidine treatment for 18 hours. While maintaining thymidine treatment, cells were subsequently treated for 24 hours with DMSO, Z-VAD-FMK (10 μM), prexasertib (100 nM),or prexasertib plus Z-VAD-FMK (10 μM). Cell viability was quantified using Cell-Titer-Glo. n=4 replicates per condition. Data are representative of two independent experiments. Multiple comparisons were analyzed by 1-way ANOVA with Tukey’s post hoc multiple comparisons test, with selected comparisons shown. *P* values of less than or equal to 0.05 were considered significant. (C) The osteosarcoma cell line U2OS with doxycycline-inducible expression of the EWS::FLI1 oncogene were treated with vehicle or doxycycline for 24 hours to induce expression of EWS::FLI1. Then, the cells were treated with thymidine (2 mM, hours 0-22) or thymidine (2 mM, hours 0-22) with prexasertib (100 nM, hours 18–22).

**Supplemental Figure 6.** (A) Ewing sarcoma cell lines were synchronized in S-phase using thymidine treatment for 18 hours. While maintaining thymidine treatment, cells were subsequently treated for 24 hours with DMSO, Z-VAD-FMK (10 μM), prexasertib (100 nM), emricasan (10 μM), prexasertib plus Z-VAD-FMK, or prexasertib plus emricasan. Cell viability was assessed using the CellTiter-Glo assay. n=4 replicates per condition. Multiple comparisons were analyzed by 1-way ANOVA with Tukey’s post hoc multiple comparisons test, with selected comparisons shown. *P* values of less than or equal to 0.05 were considered significant. ***P < 0.001; ****, P < 0.0001. (B) EW8 cells were treated with different doses of gemcitabine, with or without prexasertib (10 nM), for 24 hours. (C) EW8 cells were treated with different doses of hydroxyurea, with or without prexasertib (10 nM), for 24 hours.

**Supplemental Figure 7.** (A) EW8 cells were synchronized in S-phase using thymidine treatment for 18 hours. While maintaining thymidine treatment, cells were subsequently treated for 4 hours with DMSO, prexasertib (100 nM), NSC663284 (1 μM), or prexasertib plus NSC663284. (B) EW8 cells were treated with thymidine (2 mM, hours 0-22) followed by prexasertib (100 nM, hours 18-22), dinaciclib (1 μM, hours 18-22), or prexasertib (100 nM, hours 18-22) and dinaciclib (1 μM, hours 18-22).

**Supplemental Figure 8.** (A) Ewing sarcoma cells were synchronized in S-phase using thymidine treatment for 18 hours. While maintaining thymidine treatment, cells were subsequently treated for 4 hours with DMSO, ceralasertib (ATR inhibitor; 500 nM), Z-VAD-FMK (10 μM), or ceralasertib plus Z-VAD-FMK. (B) Ewing sarcoma cells were synchronized in S-phase using thymidine treatment for 18 hours. While maintaining thymidine treatment, cells were subsequently treated for 4 hours with DMSO, RP-6306 (PKMYT1 inhibitor; 500 nM), Z-VAD-FMK (10 μM), or RP-6306 plus Z-VAD-FMK. (C) Ewing sarcoma cells were synchronized in S-phase using thymidine treatment for 18 hours. While maintaining thymidine treatment, cells were subsequently treated for 4 hours with DMSO, prexasertib, or prexasertib in combination with either navitoclax or venetoclax. (D) EW8 and TC71 cells were synchronized in S-phase using thymidine treatment for 18 hours. While maintaining thymidine treatment, cells were subsequently treated for 24 hours with DMSO, prexasertib (100 nM), or prexasertib (100 nM) with navitoclax (1 μM). Cell viability was then quantified using Cell-Titer-Glo. Multiple comparisons were analyzed by 1-way ANOVA with Tukey’s post hoc multiple comparisons test, with selected comparisons shown. *P* values of less than or equal to 0.05 were considered significant. ****, P < 0.0001.

**Supplemental Figure 9.** (A) Cleaved caspase-8 expression (RPPA) in Ewing sarcoma cells treated with DMSO, thymidine (hours 0-22), prexasertib (hours 18-22), or thymidine (hours 0-22) with prexasertib (hours 18-22). (B) Cells were treated with thymidine (hours 0-22) and then prexasertib was added for 4 hours (hours 18-22). Protein lysates were collected at 0, 2, and 4 hours after the addition of prexasertib. (C-D) EW8 and TC71 cells were synchronized in S-phase using thymidine treatment for 18 hours. While maintaining thymidine treatment, cells were subsequently treated for 24 hours with DMSO, prexasertib (100 nM), or prexasertib (100 nM) with Z-VAD-FMK (10 μM; pan-caspase inhibitor), Z-VDVAD-FMK (10 μM; caspase-2 inhibitor), Z-IETD-FMK (10 μM; caspase-8 inhibitor), Z-LEHD-FMK (10 μM; caspase-9 inhibitor), or Z-AEVD-FMK (10 μM; caspase 10 inhibitor). Cell viability was then quantified using Cell-Titer-Glo. n=4 replicates per condition. Data are representative of two independent experiments. Multiple comparisons were analyzed by 1-way ANOVA with Tukey’s post hoc multiple comparisons test. *P* values of less than or equal to 0.05 were considered significant. ***P < 0.001.

**Supplemental Figure 10.** Ewing sarcoma cells were synchronized in S-phase using thymidine treatment for 18 hours. While maintaining thymidine treatment, cells were subsequently treated for 4 hours with DMSO, prexasertib (100 nM), Z-VAD-FMK (10 μM), or prexasertib plus Z-VAD-FMK. O-propargyl-puromycin was added for the last hour of drug incubation to label newly synthesized proteins. Puromycin was then detected by flow cytometry (Click-iT chemistry).

**Supplemental Figure 11.** EW8 and TC71 cells were treated with gemcitabine, prexasertib, or the combination of gemcitabine, prexasertib, and Z-VAD-FMK for 4 hours. Puromycin was added for the last hour of drug incubation to label newly synthesized proteins. Puromycin was then detected by immunoblotting. (B) Ewing sarcoma cell lines were treated with TAK931 (CDC7 inhibitor), AZD1775 (WEE1 inhibitor), or the drug combination for four hours. Puromycin was added for the last hour of drug incubation to label newly synthesized proteins. (C) Flow cytometry plots showing phospho-Histone H3 (p-HH3) staining in EW8 cells treated with the drugs as described in (B), with or without cycloheximide.
