# Supplementary material for: DNA replication stress and translational repression converge to drive CDK1- and caspase-dependent apoptosis in Ewing sarcoma
Source: Oncogene. 2026 Jun 10;45(28):2823–39. doi: 10.1038/s41388-026-03845-2 (PMC13337492; doi:10.1038/s41388-026-03845-2)

Supplemental Figure 1

A

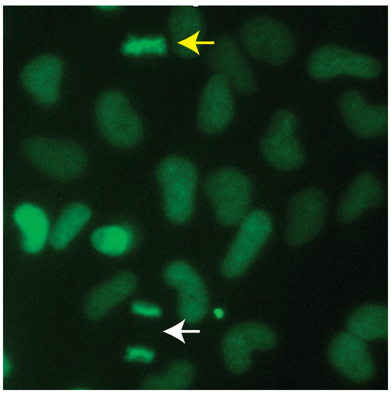

B

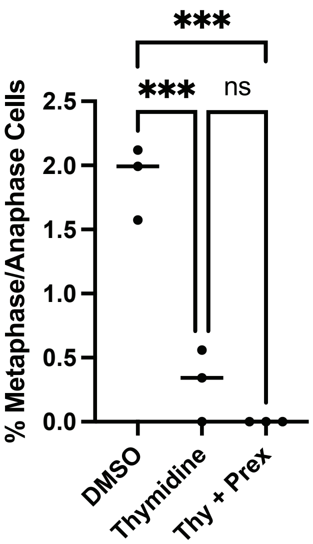

C

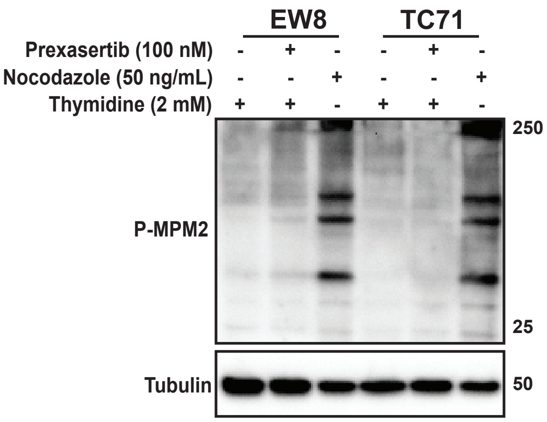

D

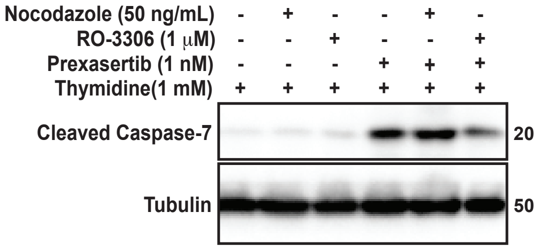

Supplemental Figure 2

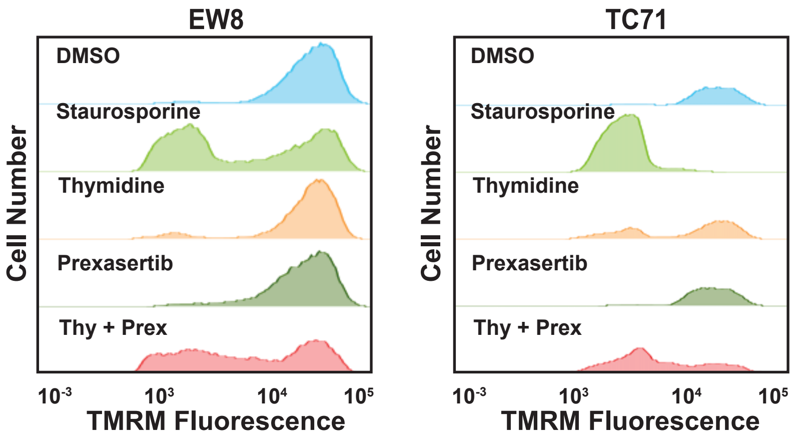

Supplemental Figure 3

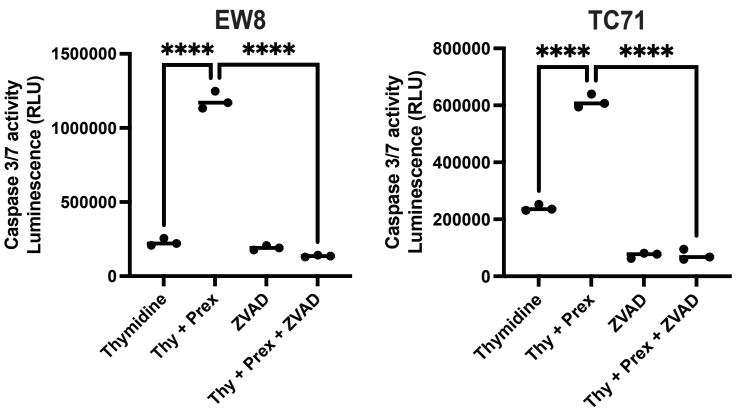

Supplemental Figure 4

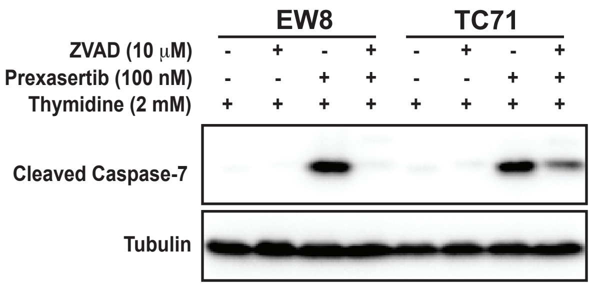

Supplemental Figure 5

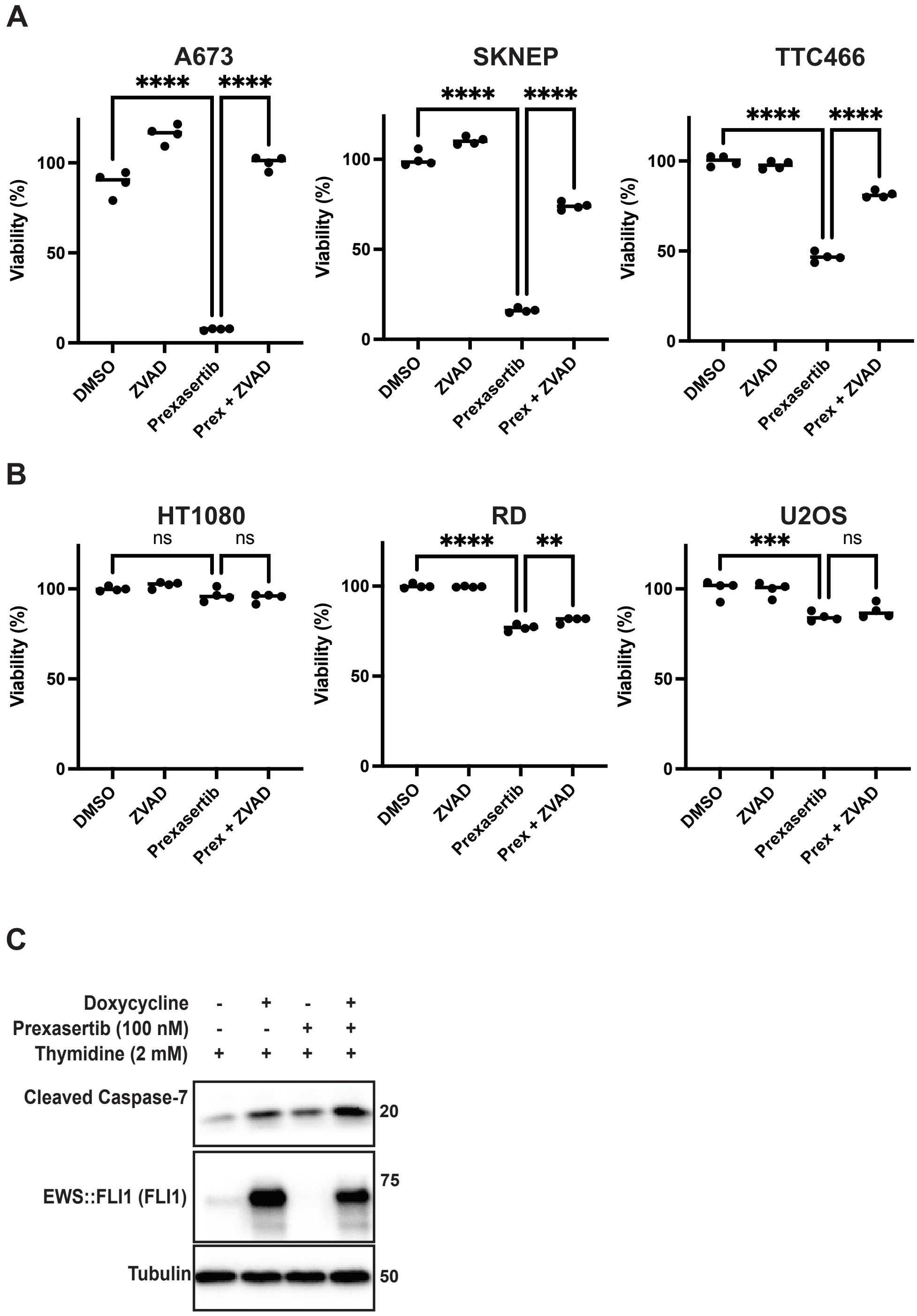

Supplemental Figure 6

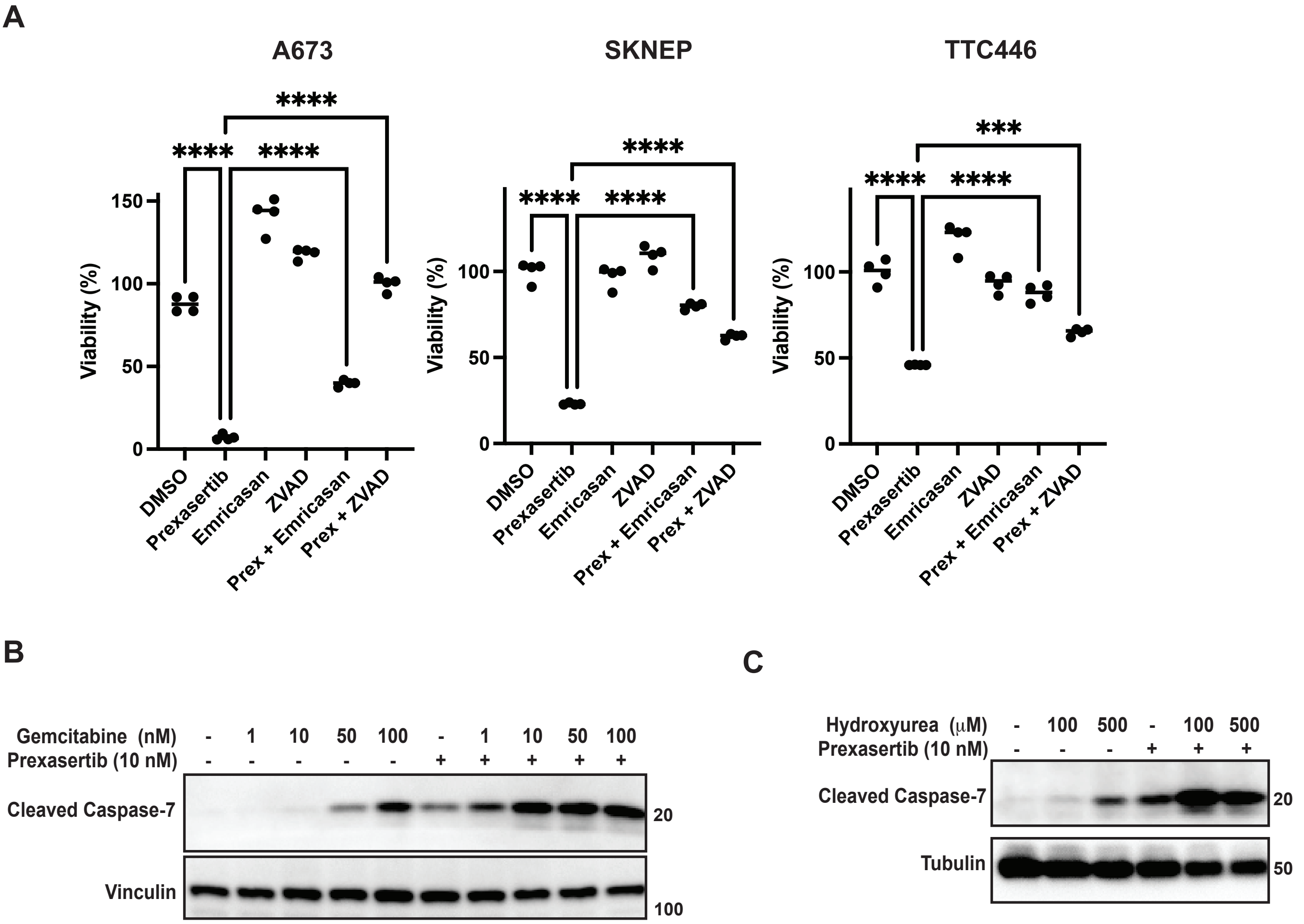

Supplemental Figure 7

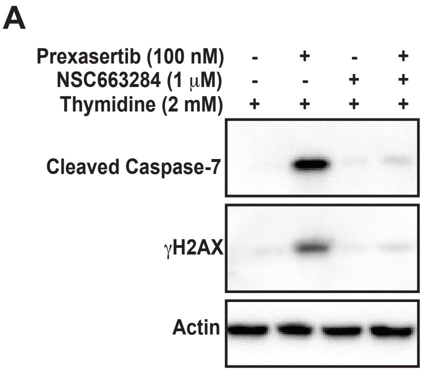

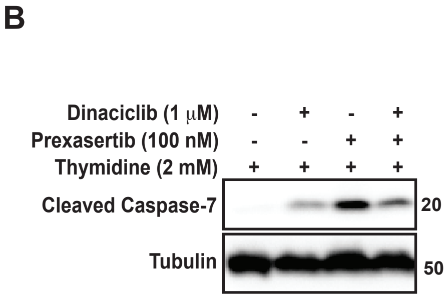

Supplemental Figure 8

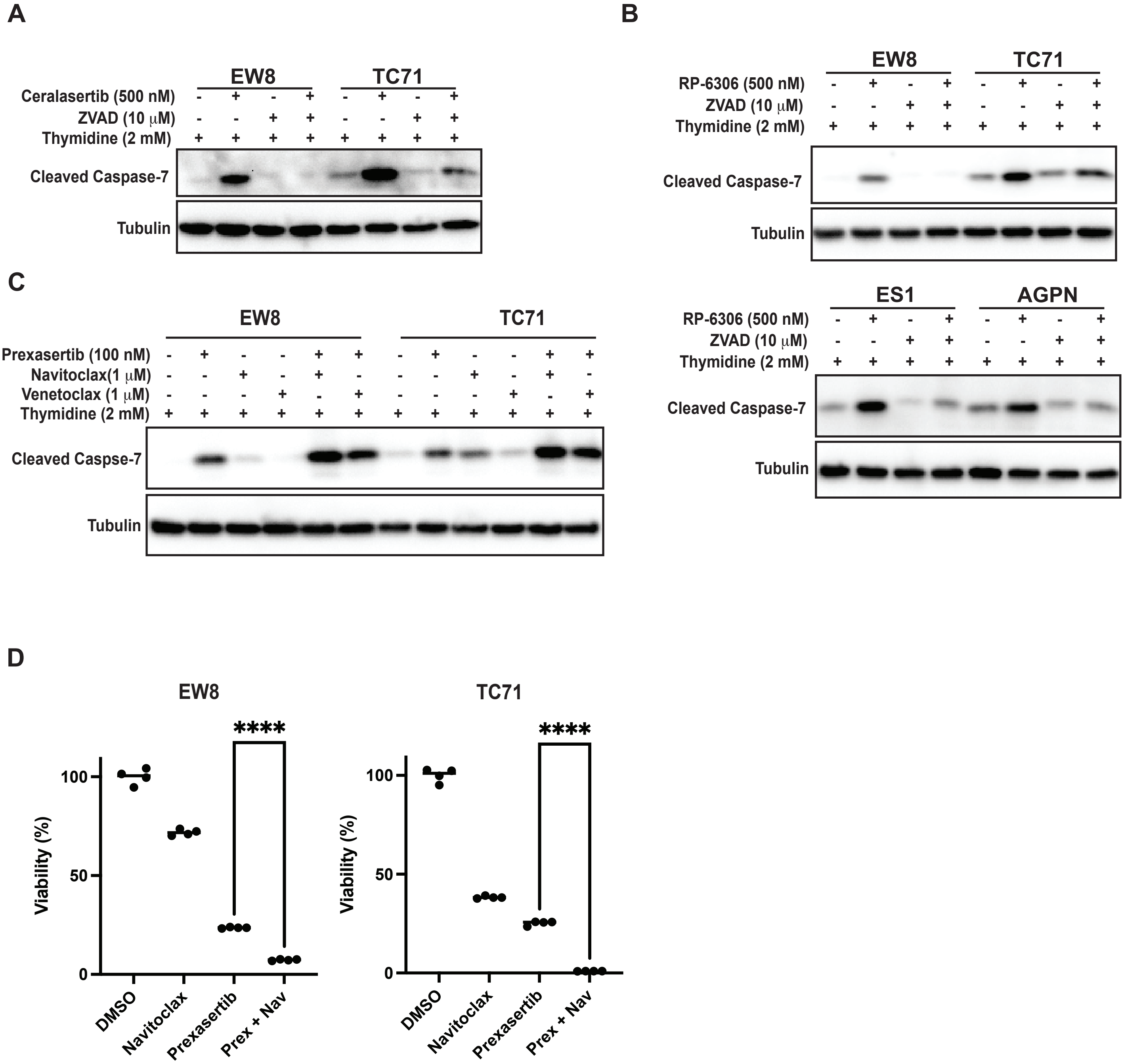

Supplemental Figure 9

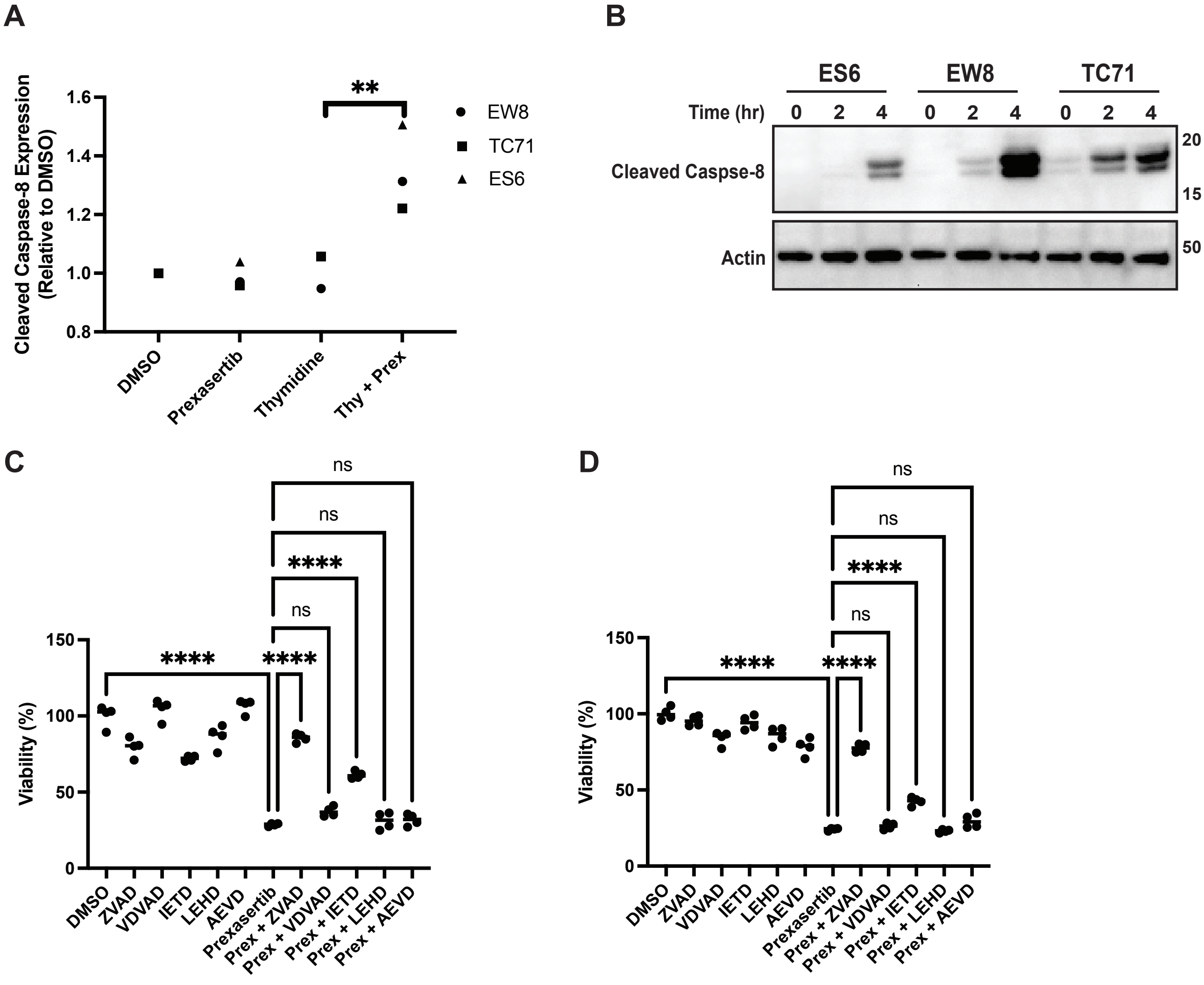

Supplemental Figure 10

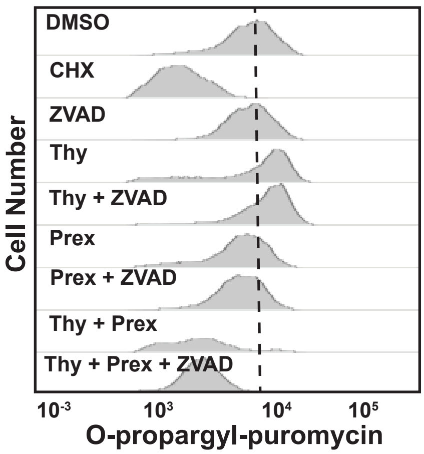

Supplemental Figure 11

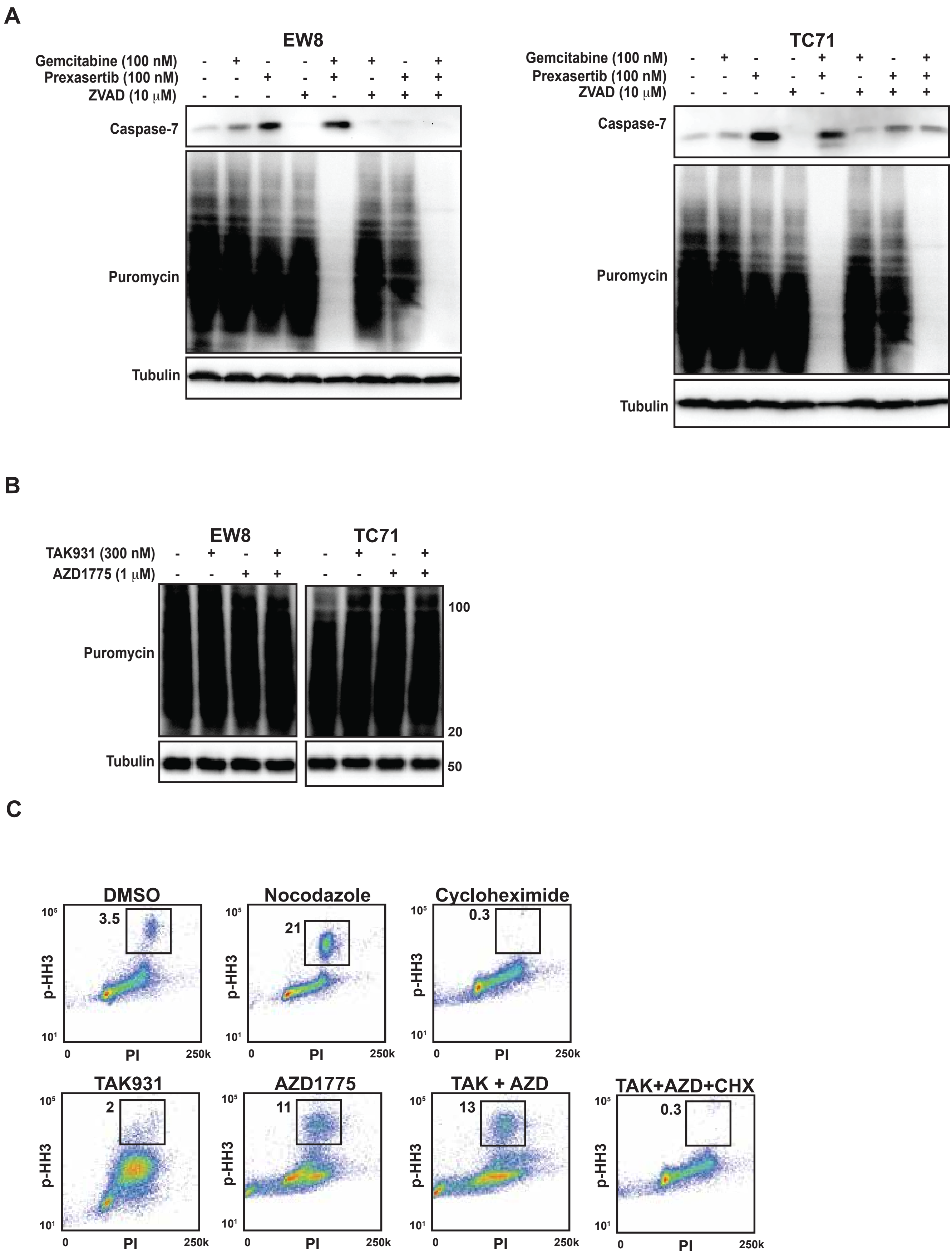

Supplement: Supplementary file 3 — Supplemental Figures [file 41388_2026_3845_MOESM3_ESM.pdf]
